# The impact of COVID-19 lockdown on child and adolescent mental health: systematic review

**DOI:** 10.1007/s00787-021-01856-w

**Published:** 2021-08-18

**Authors:** Urvashi Panchal, Gonzalo Salazar de Pablo, Macarena Franco, Carmen Moreno, Mara Parellada, Celso Arango, Paolo Fusar-Poli

**Affiliations:** 1 Child and Adolescent Mental Health Services, South London and the Maudsley NHS Foundation Trust, London, UK; 2 Early Psychosis: Interventions and Clinical-Detection (EPIC) Lab, Department of Psychosis Studies, Institute of Psychiatry, Psychology & Neuroscience, King’s College London, London, UK; 3 Institute of Psychiatry and Mental Health. Department of Child and Adolescent Psychiatry, Hospital General Universitario Gregorio Marañón, School of Medicine, Universidad Complutense, Instituto de Investigación Sanitaria Gregorio Marañón (IiSGM), CIBERSAM, Madrid, Spain; 4 Department of Psychiatry, Hospital Dr Rodríguez Lafora, Universidad Autónoma Madrid, Madrid, Spain; 5 Department of Brain and Behavioral Sciences, University of Pavia, Pavia, Italy; 6 OASIS Service, South London and Maudsley National Health Service Foundation Trust, London, UK; 7 National Institute for Health Research, Maudsley Biomedical Research Centre, South London and Maudsley NHS Foundation Trust, London, UK; 8 Department of Child and Adolescent Psychiatry, Institute of Psychiatry, Psychology and Neuroscience, PO63, 16 De Crespigny Park, London, SE5 8AF UK

**Keywords:** COVID-19, Coronavirus, Lockdown, Children, Adolescents, Mental health, Systematic review

## Abstract

COVID-19 was declared a pandemic in March 2020, resulting in many countries worldwide calling for lockdowns. This study aimed to review the existing literature on the effects of the lockdown measures established as a response to the COVID-19 pandemic on the mental health of children and adolescents. Embase, Ovid, Global Health, PsycINFO, Web of Science, and pre-print databases were searched in this PRISMA-compliant systematic review (PROSPERO: CRD42021225604). We included individual studies reporting on a wide range of mental health outcomes, including risk and protective factors, conducted in children and adolescents (aged ≤ 19 years), exposed to COVID-19 lockdown. Data extraction and quality appraisal were conducted by independent researchers, and results were synthesised by core themes. 61 articles with 54,999 children and adolescents were included (mean age = 11.3 years, 49.7% female). Anxiety symptoms and depression symptoms were common in the included studies and ranged 1.8–49.5% and 2.2–63.8%, respectively. Irritability (range = 16.7–73.2%) and anger (range = 30.0–51.3%), were also frequently reported by children and adolescents. Special needs and the presence of mental disorders before the lockdown, alongside excessive media exposure, were significant risk factors for anxiety. Parent–child communication was protective for anxiety and depression. The COVID-19 lockdown has resulted in psychological distress and highlighted vulnerable groups such as those with previous or current mental health difficulties. Supporting the mental health needs of children and adolescents at risk is key. Clinical guidelines to alleviate the negative effects of COVID-19 lockdown and public health strategies to support this population need to be developed.

**Supplementary Information:**

The online version contains supplementary material available at 10.1007/s00787-021-01856-w.

## Introduction

In January 2020, WHO first identified the novel coronavirus (COVID-19), later declaring the spread of COVID-19 as a global pandemic in March 2020 [29]. Subsequently, many countries imposed national lockdowns, closing schools and workplaces, leaving people to learn virtually, enforcing social distancing measures, and implementing restrictive measures that prevented individuals from going to public places or from meeting people from other households [45].

Quarantines and lockdowns are states of isolation that are psychologically distressing and unpleasant for anyone who experiences them [14, 50]. Young people, who are at higher risk of developing mental health problems than adults [32], may be particularly vulnerable to the adverse effects of isolation, including school closures, due to the disruption lockdown causes on their physical activity and social interaction [101].

Previous systematic reviews and meta-analyses have looked at the impact of COVID-19 on the mental health of the general population [108] and healthcare workers [31]. One previous systematic review looked at the psychological burden of quarantine associated with exposure to contagious diseases on children and adolescents but included only three articles on COVID-19 [49]. To our knowledge, this is the first comprehensive systematic review focusing exclusively on the impact of the COVID-19 pandemic response lockdown on child and adolescent mental health.

This systematic review aims to summarise the literature exploring the effects of COVID-19 lockdown on a wide range of mental health outcomes in children and adolescents. We further explore the risk factors and protective factors for developing mental health outcomes in the context of COVID-19 lockdown.

## Methods

The format of the methods and results was based on the Preferred Reporting Items for Systematic Reviews and Meta-Analyses (PRISMA) guidelines [70] (eTable 1) (study protocol registered on PROSPERO: CRD42021225604).

### Search strategy and selection criteria

A systematic search was conducted by two independent researchers (UP, MF) on Embase, Ovid MEDLINE (R), Global Health, Web of Science, and APA PsycINFO from inception until the 1st of April 2021. The search terms used can be found in eMethods 1. As this is an emerging topic, we looked at medRxiv, psyArXiv, and bioRxiv pre-print databases to identify further relevant studies. A manual search of the references of the included studies and reviews related to this topic was conducted using Google Scholar. Articles identified were screened as abstracts. After excluding those that did not meet our inclusion criteria, the full texts of the remaining articles were assessed for eligibility and decisions were made regarding their final inclusion in the review.

The inclusion criteria were as follows: (1) individual studies with original data, including grey literature, (2) conducted in children and adolescents aged ≤ 19 years, (3) exposed to COVID-19 lockdown, as operationalised in each study (see eTable 2), (4) evaluating mental health outcomes (see eTable 3 for the full list of outcomes), (5) in English. The exclusion criteria were as follows: (1) conference proceeding, abstracts, case reports or reviews, (2) studies including adults > 19 years, (3) studies in which children and adolescents were not exposed to COVID-19 lockdown, (4) studies focusing on physical health outcomes only.

### Data extraction

Independent researchers (UP, MF) carried out data extraction. Any discrepancies arising were resolved through consensus, consulting another researcher (GSP) if an agreement was not attained. The variables extracted included: lead author/year, country, study design (cross-sectional, cohort, qualitative, mixed methods), sample size, sex (% females), age (mean ± SD, range), exposure data (lockdown definition, length of lockdown), instruments, outcomes (see eTable 3), report (parent, children), quality appraisal (see below) and key findings.

### Strategy for data synthesis

The results of the systematic review were summarised in tables and narratively synthesised. Results were stratified by poor mental health outcomes and risk factors, followed by good mental health outcomes and protective factors.

### Quality appraisal

For study appraisal, this review used the Newcastle–Ottawa Scale (NOS) adapted for cross-sectional studies [69], which has been attached as a supplementary file (eMethods 2). This scale has three domains: selection, comparability, and outcome. The domain of selection has four categories assessing the representativeness of the sample, the sample size, the number of non-respondents, and the ascertainment of the exposure, with a maximum of five stars to be awarded. The domain of comparability has one category assessing if confounding factors are controlled for, with the maximum award of two stars. The final domain of outcome has two categories assessing the outcome and the appropriate usage of statistical tests, with the maximum award of three stars. All categories can score one star, apart from the ascertainment of the exposure and assessment of the outcome, both of which can score two stars. A total of 10 stars can be awarded if a study meets all the criteria specified.

## Results

### Search results

**Fig. 1:**
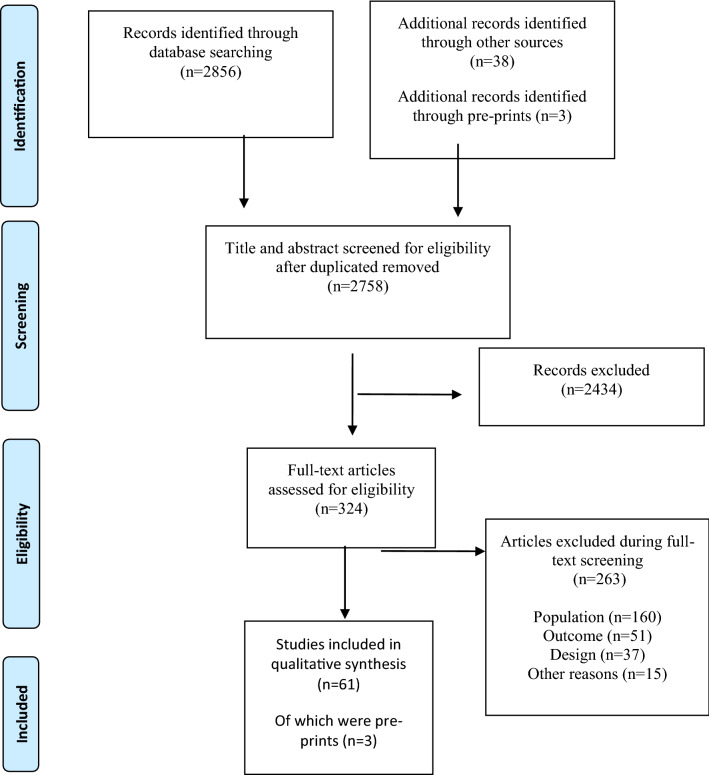
PRISMA flowchart

A systematic electronic search identified a total of 2856 publications and 41 additional articles were found via backward searching of key papers. Of those, 324 publications underwent full-text screening. A total of 263 publications were excluded at the full-text screening stage and 61 articles finally met the criteria for inclusion, 3 of which were identified in the pre-print databases. Results of the search follow in the PRISMA 2009 flow diagram (Fig. 1).

### Study characteristics

The sample sizes within the included studies ranged 15–7,772 participants, (n = 54,999). The mean age of participants was 11.3 years (range: 1–19 years). 49.7% of participants were female. Most studies were cross-sectional studies (*n* = 45, 73.8%) while the rest were longitudinal studies (*n* = 16, 26.2%). Included studies took place across five continents including Europe (*n* = 35, 57.4%), Asia (*n* = 22, 36.1%), Australia (*n* = 1, 1.6%), North America (*n* = 1, 1.6%), South America (*n* = 1, 1.6%), and across more than one continent (*n* = 1, 1.6%). Most studies involved only parent self-reports (*n* = 21, 18,655) or solely child self-reports (*n* = 20, 25,327), while other studies involved both parent and child self-reports (*n* = 10, 7,931). A proportion of studies (*n* = 10, 2,321) utilised interviews, of which some were parent interviews (*n* = 2, 535), some were child interviews (n = 5, 2,629), and some interviewed both parents and children (*n* = 3, 261). Duration of lockdown was 52.3 ± 21.3 days in the included studies (range 30 [1, 4, 12, 17, 25, 27, 34, 39, 41, 57, 82]—100 days [80, 100]). The characteristics of the included studies and their findings are summarised in Table 1.

**Table 1:** Characteristics of the included studies

Lead Author/year	Country	Study design	Sample size	Sex (*F*%)	Age (Mean ± SD, Range)	School closure mentioned	Length of lockdown	Key findings
Abawi et al. 2020 [1]	Netherlands	Cross-sectional	75	52%	10.5, 7–15		★★	32.0% of children reported COVID-19 related anxiety. 25.0% of the families imposed their own quarantine measures. Many of the included families found that previous service contact helped to alleviate anxiety
Abdulah et al. 2020 [2]	Iraqi Kurdistan	Cross-sectional	15	53%	6–13		N.A	Being at home during the COVID-19 outbreak was seen to result in high levels of stress in children. Children expressed fear about coronavirus. Due to home confinement and social distancing, children also experienced loneliness, stress, sadness, and depression
Achterberg et al. 2020 [3]	Netherlands	Longitudinal	151		10–13		N.A	Children’s externalising behaviour changes were mediated by perceived stress (*p* < 0.001): higher scores before lockdown were related to higher stress during the lockdown, Perceived stress in children was associated with negative coping strategies (*p* = 0.006). Children’s stress levels were influenced by prior and current parental over-reactivity (*p* = 0.001)
Adibelli et al. 2020 [4]	Turkey	Cross-sectional	597	56%	9.9 ± 2.0, 7–13		★★	The emotional well-being (*p* < 0.001), self-esteem (*p* < 0.001), family (*p* < 0.01), school (*p* < 0.05) sub-dimensions and total (*p* < 0.05) scores of children who tended to use the internet were found to be lower. Emotional well-being (*p* < 0.001), family (*p* < 0.01), friends (*p* < 0.05) sub-dimensions of the children of the parents who feel fear/anxiety about coronavirus becoming a pandemic were lower
Alves et al. 2020 [5]	America	Longitudinal	64	63%	11.8 ± 1.3, 9–15	Y	★	Positive affect was associated with lower state anxiety, even when adjusting for child age, sex, SES, and BMI z-scores (*β* = − 0.40, *p* < 0.001). Negative effect was correlated with sedentary time (*r* = 0.28, *p* = 0.02) and leisure screen time (*r* = 0.40, *p* = 0.001)
Amorim et al. 2020 [6]	Portugal	Cross-sectional	99	69%	10.8 ± 3.1		N.A	72.1% of parents reported a change in behaviour in children with ASD compared to 32.1% in the control group (*p* < 0.05). The changes of behaviour in children with ASD were reported to be due to anxiety (41.7%), irritability (16.7%), obsessions (11.1%), hostility (5.6%), and impulsivity (2.8%). Children with ASD and their parents reported higher anxiety levels compared to controls (*p* < 0.05)
Asanov et al. 2021 [7]	Ecuador	Cross-sectional	1320	53%	15.9, 14–18	Y	★★★	16.0% of students have mental health scores that are indicative of depression. School closure and social isolation are the key stressors identified by students
Baptista et al. 2020 [10]	Portugal, Brazil	Cross-sectional	253	48%	7.5, 3–15	Y	★★★	72.2% of parents report changes in their child's routine during social distancing. Sleep breathing disorders (*p* = 0.019), sleep–wake transition disorders (*p* = 0.022) were reported
Bentenuto et al. 2021 [11]	Italy	Cross-sectional	164	26%	3–17		N.A	Significant increases in child's externalising behaviours were seen. In children with NDDs, the decrease in therapeutic/rehabilitation support predicted higher externalising behaviours
Bignardi et al. 2020 [12]	UK	Longitudinal	168	55%	7.6–11.6	Y	★★	Children’s depressive symptoms increased (*p* < 0.001), as relative to before lockdown. Non-significant, small changes were seen in anxiety and emotional problems
Cauberghe et al. 2020 [16]	Belgium	Cross-sectional	2165	67%	15.5 ± 1.6, 13–19	Y	N.A	Adolescents who were anxious used social media to adapt to COVID-19 more than as a method of keeping in contact with friends and family (*p* < 0.001). The indirect effect of anxiety was seen to have a significantly positive effect on happiness via active coping (*p* < 0.0016). Those who felt lonelier were more likely to use social media to deal with the lack of social contact (*p* = 0.004). Humorous coping was found to be positively related to feelings of happiness and not influenced by anxiety or loneliness (*p* = 0.008)
Cetin et al. 2020 [17]	Turkey	Cross-sectional	76	30%	10.1 ± 2.2	Y	★★	Sleep problems mediated the relationship between PTSD symptoms and severity of ADHD symptoms and the relationship between chronotype and the severity of ADHD symptoms
Chen et al. 2020a [19]	China	Longitudinal	543	51%	10.9 ± 0.7	Y	N.A	At follow-up there were greater levels of psychological distress for school children. A significant predictor of psychological distress at baseline and follow up was seen to be problematic internet-related behaviours. Other significant predictors for psychological distress at follow up were follow up illness status, perceived academic performance, and problematic smartphone-app usage (*p* < 0.001)
Chen et al. 2020b [18]	China	Cross-sectional	1036	49%	6–15	Y	N.A	11.8% of participants showed depression, 18.9% of participants showed anxiety and 6.6% of participants showed anxiety and depression. Female adolescents showed higher risk of depression and anxiety during COVID-19. Adolescents (13–15 years) were seen to be more depressed than younger children
Chen et al. 2020 [20]	China	Cross-sectional	7772	52%	12–18		N.A	A significant difference was seen in anxiety symptoms for participants who were from Wuhan compared to other urban areas (*p* = 0.004). Participants’ gender, a relative being infected, and online education were seen to have direct positive predictive value for depressive and anxiety symptoms (*p* < 0.001). Having relatives who participated in COVID-19 related work predicted developing depressive symptoms (*p* < 0.05)
Commodari et al. .2020 [25]	Italy	Cross-sectional	978	65%	16.6 ± 1.2, 13–20		★★	Females showed less self-confidence levels than males (*p* < 0.001). Students reported feeling tense (40.0%), sad (42.6%), and irritable (49.6%). 55.9% reported difficulties concentrating and 55.6% reported difficulties sleeping. 13.4% reported eating difficulties where they forgot to eat or skipped meals. 18.7% reported disturbances in heartbeat (18.7%). Significant predictors of negative feelings were female gender (*β* = 0.284, *p* < 0.001), age (β = 0.119 *p* < 0.001), living in a red zone (*β* = 0.090, *p* = 0.004), perceived seriousness (*β* = 0.085, *p* = 0.007), fear of getting COVID-19 (*β* = 0.091, *p* = 0.005), and compliance with government measures (*β* = 0.152, *p* = 0.001)
Conti et al. 2020 [27]	Italy	Longitudinal	141	17%	1.5—18		★★	Within the 1.5–5-year-old population, anxiety (*p* < 0.05) increased. Within the 6–18-year-old population, obsessive–compulsive (*p* < 0.05) and thought problems increased (*p* < 0.05). In the regression models, younger age in the 1.5–5-year-old population was seen as “protective” (*p* < 0.05). During lockdown, familial financial hardship was associated with an increase in psychiatric symptoms in the 6–18-year-old population (*p* < 0.05)
Cusinato et al. 2020 [30]	Italy	Cross-sectional	463	44%	9.7 ± 3.3, 5–17	Y	★★	Females obtained higher prosocial behaviour scores than boys (*p* < 0.001)
Di Giorgio et al. 2020 [34]	Italy	Cross-sectional	245	48%	4.1, 2–5		★★	Children went to bed on average ~ 53 min later (*p* < 0.0001) and woke up ~ 66 min later (*p* < 0.0001) during the lockdown. An increase in emotion symptoms (*p* = 0.011), conduct problems (*p* = 0.003) and hyperactivity/inattention issues (*p* < 0.0001) was seen in children during the lockdown
Ezpeleta et al. 2020 [38]	Spain	Longitudinal	226	52%	13.9 ± 0.3	Y	★★	The mental health of adolescents’ during the COVID-19 lockdown was associated with the activities and routines adolescents' kept up (*p* = 0.005), the quality of their relationships with friends (*p* = 0.001), parents (*p* < 0.001), and siblings (*p* = 0.006), how adults around them were affected by the lockdown (*p* = 0.002), the physical environment in which they were locked down (*p* = 0.023), how they reacted to the lockdown in terms of feelings and behaviours (*p* = 0.017), and how the disease affected the immediate family (*p* = 0.002)
Francisco et al. 2020 [39]	Italy, Spain, Portugal	Cross-sectional	1480	47%	9.2 ± 4.3, 3–18		★★	Approximately one-third of children report being restless, nervous, worried, uneasy, lonely, and anxious. 52.2% report being bored and > 40% irritable. The mean number of hours of sleep during weekdays significantly increased during home confinement for the total sample (*p* < 0.001, *r* = 0.30)
Giannopoulou et al. 2021[41]	Greece	Cross-sectional	442	68%	16–18	Y	★★	The prevalence of a positive screen for depression (PHQ-9 score ≥ 11) rose from 48.5% to 63.8% where those scoring within the severe depression range (PHQ-9 ≥ 20) rose from 10 to 27%. The prevalence of a positive screen for anxiety (GAD-7 score ≥ 11) rose from 23.8% to 49.5%, where those scoring within severe anxiety range (GAD-7 ≥ 17) rose from 3.8% to 20.5%
Gimenez-Dasi et al. 2020 [42]	Spain	Longitudinal	167	42%	7.0 ± 2.6. 3–11		★★	Significant differences were seen in the attention (*p* = 0.02), willingness to study (*p* < 0.001), emotional regulation problems (*p* < 0.001), and hyperactivity and impulsivity (*p* < 0.001) scales from before to after confinement
Graell et al. 2020 [44]	Spain	Cross-sectional	365	88%	14.5 ± 2.3, 7–15		★★	41.9% of the children and adolescents experienced reactivation of eating disorder (ED) symptoms despite treatment (p = 0.005). Adolescents experienced a more pronounced reactivation of ED and non-ED symptoms than children, and severe patients (25.0%) presented a risk of self-harm and suicide. There was less weight loss monitoring in children during confinement (*p* = 0.02). On admission, 45.5% of patients presented irritability and 22.7% presented mood disturbances, due to confinement
Idoiaga et al. 2020a [47]	Spain	Cross-sectional	250	52%	7.1 ± 2.6, 3–12		★★	Lockdown was reported to result in mixed emotions in children; ranging from happy and relaxed to fear, nervousness, worry, loneliness, sadness, boredom, and anger. Children expressed difficulty due to the deprivation of fresh air and outdoor exercise in lockdown, resulting in a more sedentary state
Idoiaga et al. 2020b [48]	Spain	Cross-sectional	228	52%	7.1 ± 2.6, 3–12	Y	★★	Children experienced conflicting emotions due to lockdown as being scared, nervous, lonely, sad and angry, but also feeling safe, calm, and happy. Older children who are 6–12 years of age report more concern over the highly contagious nature of COVID-19, and experience sadness, fear, concern and nervous when asked about coronavirus
Kılınçel et al. 2020 [51]	Turkey	Cross-sectional	745	70%	16.8 ± 1.7, 12–18	Y	N.A	Young people experienced anxiety and loneliness due to the closure of schools and home-quarantine due to the pandemic (*p* = 0.001). In the group that mostly used the television as a source of information about COVID-19, the state anxiety scores were higher (OR = 2.4). Adolescents previously referred for psychiatric treatment had higher anxiety scores (OR = 4.4)
Larsen et al. 2020 [53]	Norway	Longitudinal	442	55%	11.4 ± 2.6		★★	Results showed significant associations between emotional, somatic/cognitive, and worry reactions and COVID-19 related predictors: home school experience, family stress and instability, missing friends and worry about virus infection (p < 0.001). Older children reported more negative reactions
Lecuelle et al. 2020 [54]	France	Longitudinal/Retrospective	92		29.6 months		★★	The lockdown reduced the frequency (p = 0.02) and length (p = 0.01) of naps. Nocturnal sleep duration increased (p < 0 .001). Frequency of parasomnia increased from 6.0 to 7.1 (p = 0.003)
Liang et al. 2020 [56]	Italy	Cross-sectional	1074	48%	9.0 ± 2.0, 6–12	Y	N.A	89.7% of children were affected during quarantine. The symptoms of anxiety differed significantly between the two regions (*p* < 0.001). Children in northern areas appeared to be more worried (*p* < 0.001), more preoccupied with death (*p* < 0.001), more easily alarmed (*p* < 0.01), and more afraid of COVID-19 infection (*p* < 0.001), compared to central areas. There were also significant differences seen regarding mood symptoms (*p* < .01). Children in the northern areas were sadder (*p* < 0.001) and more bored (*p* < 0.01) in comparison to children in central areas
Liebana-Presa et al. 2020 [57]	Spain	Cross-sectional	300	62	14.0 ± 1.0, 13–17		★★	There was a strong correlation between the physiological and emotional manifestations in stress (r = 0.778). Regarding the intention to use cannabis, the component of attitude toward its use is significantly correlated with stress: emotional manifestations (r = 0.260), physiological (*r* = 0.300) and behavioural (*r* = 0.412)
Liu et al. 2020 [59]	China	Cross-sectional	1264	44%	9.8, 7–12	Y	N.A	Amongst children, prosocial behaviours prevalence was 10.3%, followed by conduct problems (7.0%), peer problems (6.6%), hyperactivity-inattention (6.3%) and emotional problems (4.7%). Children who did physical activity had a lower hyperactivity-inattention risk (OR: 0.44 for 1–2 days/week; OR: 0.56 for more than 2 days/week) and less prosocial behaviours problems (OR: 0.65 for 1–2 days/week; OR: 0.55 for more than 2 days/week), compared to children who did not exercise
Magson et al. 2020 [62]	Australia	Longitudinal	248	51%	14.4 ± 0.5, 13–16	Y	N.A	Adolescents reported a significant increase in the experience of depressive symptoms (*p* < 0.001) and anxiety (*p* < 0.001), alongside a decrease in life satisfaction (*p* < 0.001) from baseline to follow-up. An increase in depression at follow-up was associated with COVID-19 related worries (*p* < 0.001), online learning difficulties (*p* < 0.001), and increased conflict with parents (*p* = 0.007). An increase in anxiety at follow up was associated with gender (*p* = 0.041), COVID-19 distress (*p* = 0.042), media (*p* = 0.035), and social disconnection (*p* < 0.001)
Majeed et al. 2020 [63]	Pakistan	Cross-sectional	63	51%	13–17	Y	N.A	Most adolescents showed symptoms of depression, anxiety, and anger. Females reported more somatic complaints (*p* = 0.01), whereas males reported more anger problems (*p* = 0.01). Severe irritability and expressions of anger were reported
Mallik et al. 2021 [64]	Bangladesh	Cross sectional	552	N.A	10.4 ± 4.1, 4–17		N.A	Pre-lockdown, females reported more emotional disorders (9.4%) than boys (5.4%) (*p* < 0.05). A significant association between boys with conduct disorder during the lockdown period (*p* < 0.05) was seen. During lockdown, the prevalence of conduct disorder among the boys and girls were 32.8% and 25.5%, respectively. Hyperactivity significantly increased amongst boys during lockdown (*p* < 0.001)
Morgul et al. 2020 [72]	UK	Cross-sectional	927	N.A	5–11	Y	★★	Children were more bored (73.8%), lonely (64.5%), sad (43.4%), frustrated (61.4%), irritable (57.1%), restless (52.9%), worried (52.4%), angry (48.6%), anxious (45.2%), and were more argumentative with the rest of the family (29.7%) during the lockdown compared to the pre-COVID-19 period. During the lockdown, children spent significantly more time using screens (*p* < 0.001), and less time doing physical activity (*p* < 0.001) and sleeping (*p* = 0.001)
Mourouvaye et al. 2020 [73]	France	Longitudinal	234	72%	13.4 ± 1.8, 7–17		★★	We found a significant decrease in the incidence of admissions for suicide behaviour during the lockdown (IRR: 0.46, 95% CI: 0.24 to 0.86)
Nonweilier et al. 2020 [74]	UK	Cross-sectional	371	29%	4–15		★★	Young people with neurodevelopmental disorders, compared to neurotypical controls, had a higher prevalence of emotional symptoms (42% vs. 15%) (*p* < 0.001) and conduct problems (28% vs. 9%) (*p* < 0.001), and fewer prosocial behaviours (54% vs. 22%) (*p* < 0.001). Participants with ADHD showed inflated conduct problems (*p* < 0.01), while participants with ASD showed decreased prosocial behaviours (*p* = 0.04). Females with ASD had higher emotional symptoms compared to males (*p* < 0.001)
Orgilés et al. 2020 [75]	Spain & Italy	Cross-sectional	1143	48%	9.1 ± 4.2, 3–18	Y	N.A	During quarantine, 85.7% of the parents reported perceived changes in their children´s emotional state and behaviours. In children, the most frequent symptoms seen were difficulty concentrating (76.6%) (*p* < 0.001), boredom (52%), irritability (39%) (*p* < 0.05), restlessness (38.8%) (*p* < 0.001), nervousness (38%) (*p* < 0.001), feelings of loneliness (31.3%) (*p* < 0.001), uneasiness (30.4%) (*p* < 0.001), and worries (30.1%)
Patra et al. 2020 [78]	India	Cross-sectional	225	38%	11.0	Y	N.A	> 90% of parents reported improvements in their child’s physical and psychological health. 30% of parents reported worsening of child behaviour regarding "anger". 3% of children worsened in the domains of "neatness ability" and "eating behaviour"
Pisano et al. 2020 [79]	Italy	Cross-sectional	5989		4–10	Y	N.A	54% of children showed increased irritability, intolerance to rules, whims and excessive demands, 21% presented mood changes and 20% reported sleep problems. 34.3% displayed nervousness when the pandemic was mentioned at home or on TV. 31% seemed calmer and 50% seemed wiser and more thoughtful. 93% seemed able to adapt to the pandemic restrictions
Pons et al. 2020 [80]	Spain	Cross-sectional	544	49%	15.9 ± 1.5		★★★	Young athletes reported higher anxious/depressive (3.5 ± 1.0) (*p* < 0.001) and social dysfunction (3.5 ± 1.5) (*p* < 0.001) symptoms. 54.8% of the sample reported a low negative impact of lockdown on life-spheres and few mental health issues. 30.0% of the participants reported a medium negative impact on life-spheres and moderate mental health issues. 15.3% of the sample showed a high negative impact of the COVID-19 lockdown with high mental health issues
Radwan et al. 2020 [82]	Palestine	Cross-sectional	942	66%	6–18	Y	★★	78.1% of students were psychologically affected. Female students were more psychologically affected, experiencing significantly greater fear than male students (*p* < 0.001). This study showed a significant positive correlation between social media and spreading panic about COVID-19 (*r* = 0.891, *p* < 0.001) and the effect of social media panic depending on a student’s age and gender (*p* < 0.001)
Ren et al. 2020 [83]	China	Cross-sectional	1487	51%	13.1 ± 1.6, 10–17	Y	★★	Adolescents’ depressive symptoms after quarantine was impacted by the presence of cases in their community during the quarantine (p < 0.001); this was especially the case in older adolescents (p < 0.001). All types of routine were associated with a decrease in adolescent depressive symptoms (p < 0.001)
Romero et al. 2020 [85]	Spain	Longitudinal	1049	50%	7.3 ± 2.4, 3–12	Y	★★	Child adjustment was influenced by parents’ perceived distress and emotional response to the COVID-19 crisis, parenting distress and specific parenting practices. Preschool children (aged 3 to 6 years old) showed a higher increase in conduct problems and hyperactivity as compared to their school-aged counterparts. Older children (aged 10 to 12 years old) showed the lowest increase in hyperactivity. Child’s conduct problems and hyperactivity were negatively affected by age (ß = − 0.10, *p* < 0.01 and ß = − 0.09, *p* < 0.05)
Sama et al. 2020 [86]	India	Cross-sectional	310	42%	Children		★★	73.1% of the children were having signs of increased irritation and 51.2% of children reported increased signs of anger; 18.7% of parents reported symptoms of depression and 17.6% of parents also mentioned the symptoms of anxiety amongst their children. These factors were also affected by the changes in the child's diet, sleep, weight, and the increased usage of the electronic equipment
Saurabh et al. 2020 [87]	India	Cross-sectional	121	15%	15.4, 9–18		N.A	Children and adolescents in quarantine experienced greater psychological distress than non-quarantined children and adolescents. The most common feelings reported during the quarantine were 69% experienced worry, 66% experienced helplessness, and 62% experienced fear
Shah et al. 2020a [89]	India	Cross-sectional	423	46%	12.3 ± 1.6, 11–15	Y	N.A	30.7% of children experienced psychosocial problems, of which, 25.2% had anxiety or depressive symptoms due to lockdown. The common reasons for which were fear of acquiring COVID-19 infection (60%), not able to attend school (56%), and not able to meet friends (80%). 5.4% of young people felt hopeless, 25.2% seemed to be having less fun, and 23.4% were feeling sad or unhappy. 24.3% reported worrying a lot and 12.5% were ‘down on oneself.’ 58% of children were happy to spend more time with family and 33% did not feel any anything unusual. Increased use of social media was associated with higher risk of anxiety or depressive symptoms [OR = 1.83, *p* = 0.001]
Shah et al. 2020b [88]	India	Longitudinal	48	10%	9.8 ± 3.7	Y	N.A	During the lockdown period, there was worsening of symptoms of ADHD shown by an increase in the activity level (50.1%), irritability (45.8%), and disturbing/disruptive behaviour (47.7%) in children. Regarding the behaviour of family members, there was increase in irritability (37.5%), and shouting at the child (43.8%), verbal abuse (25%), and punishing the child (27.1%). Additionally, there was an increase in praising (67.6%) and spending time with the child (72.9%). Children also reported an increase in anxiety (29.1%), feelings of boredom (35.4%), demanded more time from the parents (35.5%), and were distressed for not being able to go out of the home (39.6%)
Smirni et al. 2020 [90]	Italy	Cross-sectional	148	57%	17.9 ± 1.2, 17–19		N.A	Anxiety scores were high for older adolescents during the COVID-19 pandemic. > 50% items on the SAS tool reached a high anxiety score. The most anxiety-provoking symptom for older adolescents was breathing difficulties
Spinelli et al. 2020 [91]	Italy	Cross-sectional	854	50%	7.1 ± 3.4, 2–14		★★	Quarantine’s impact on children’s behavioural and emotional problems is mediated by parent’s individual and dyadic stress. Parent stress is significantly correlated with hyper-inattention in children (R:0.44, *p* < 0.001)
Tang et al. 2020 [94]	China	Cross-sectional	4342	49%	11.9 ± 2.3, 6–17		★★	Anxiety (24.9%), depression (19.7%), and stress (15.2%) were common during the pandemic. Children who had discussions with their parents about COVID-19 experienced less depression (*p* < 0.001), anxiety (*p* < 0.001), and stress (*p* < 0.001)
Troncone et al. 2020 [96]	Italy	Cross-sectional	414	57%	13 ± 3, 8–19	Y	★★	8.7% of participants with Type 1 diabetes and 13.4% of controls had scores indicating the disordered eating behaviours. Female gender (*p* < 0.0001) was found to be a significant predictor of disordered eating behaviours
Waite et al. 2020 [100]	UK	Longitudinal	2673	48%	4–16		★★	Pre-adolescent children exhibited a deterioration in mental health symptoms resulting in a 10% increase in meeting probably caseness criteria for emotional symptoms, a 20% increase in hyperactivity/inattention and a 35% increase in conduct problems. Changes amongst adolescents were smaller, resulting in a 4% increase in hyperactivity/inattention, 8% increase in conduct problems, and a 3% reduction in caseness criteria for emotional symptoms. Children and adolescents in low-income households, those with special educational needs and/or neurodevelopmental disorders, exhibited elevated symptoms and caseness at both time points
Wiguna et al. 2020 [103]	Indonesia	Cross-sectional	113	47%	14.7 ± 2.2, 11–17	Y	★★	The number of adolescents that perceived their own significantly worsening mental wellbeing increased during COVID-19 pandemic, in comparison to before the pandemic (*p* < 0.05). There were significant associations between having mental health information and conduct behaviour (OR: 10.34, 95% CI: 1.27–78.86); Subjective anxiety due to COVID-19 pandemic and pro-social behaviour problems (OR: 2.37, 95% CI: 1.00–5.63), parental support and total difficulties (OR: 0.09, 95% CI: 0.14–0.60) and pro-social behaviour problems (OR: 0.09, 95% CI: 0.01–0.82); friends support during COVID-19 pandemic and conduct behaviour (OR: 0.20, 95% CI: 0.04–1.00)
Xiang et al. 2020 [106]	China	Longitudinal	2427	49%	6–17	Y	★★	Mean depression scores significantly decreased during school closure (*p* < 0.01). Children in middle school showed a greater decrease in depression scores than those in primary school (*p* = 0.09), whereas a lower depression score during school closure was consistently observed across sexes and household income categories
Xie et al. 2020 [107]	China	Cross-sectional	1784	43%	7–12	Y	★★	23% of students reported depressive symptoms and 19% of anxiety symptoms. Students in Wuhan had more severe depressive symptoms than students in Huangshi (*p* = 0.02). Students who experienced slight to no worry about being affected by COVID-19 had lower depressive symptoms than those who reported higher worry (*p* < 0.001). Those reporting not being optimistic about COVID-19 had more severe depressive symptoms (*p* < 0.001)
Yeasmin et al. 2020 [109]	Bangladesh	Cross-sectional	384		5–15	Y	N.A	43% of children were found to have met the subthreshold for depression, anxiety, and sleep disorder. 31% of children experienced mild disturbances regarding depression, anxiety, and sleeping disorder. 19% reported a moderate mental disturbance regarding depression, anxiety, and sleeping (*p* < 0.001). 7% reported a severe mental disturbance regarding depression, anxiety, and sleeping disorder. Mental disturbances in children were correlated with parent's stress and abnormal behaviour
Yue et al. 2020 [110]	China	Cross-sectional	1360	46%	10.6		N.A	1.8% of children experienced moderate anxiety, 2.2% reported depression, and 3.2% of children met diagnostic criteria for PTSD. For children, excessive media exposure was a risk factor for anxiety and PTSD (*p* < 0.05)
Zhang et al. 2020 [111]	China	Longitudinal	1241	42%	12.6 ± 1.4, 9–16	Y	★★	The prevalence of mental health outcomes among students in May 2020 after lockdown increased significantly from levels early on in the pandemic in November 2019: depressive symptoms (24.9% vs 18.5%; OR:1.50, *p* = .001), nonsuicidal self-injury (42.0% vs 31.8%; OR: 1.35, *p* < .001), suicide ideation (29.7% vs 22.5%; OR: 1.32, *p* = .008), suicide plan (14.6% vs 8.7%; OR: 1.71, *p* < .001), and suicide attempt (6.4% vs 3.0%; OR: 1.74, *p* < .001)
Zijlmans et al. 2020 [112]	Netherlands	Cross-sectional	1183		8–18	Y	★★	The psychiatric sample reported significantly more problems than the general population sample on all measures except anxiety and peer relationships (*p* < 0.05). A friend or relative affected by COVID-19 and a COVID-19 related change in work situation negatively moderated outcomes and was associated with more anxiety and depressive symptoms (*p* < 0.01). Higher age was significantly associated with higher anxiety (*p* < 0.01). Male gender was significantly associated with lower anxiety and depressive symptoms (*p* < 0.01)

*SES* socio-economic status, *BMI* body mass index, *ASD* autism spectrum disorder, *NDD* neuro-developmental disorder, *COVID-19* coronavirus-19, *PTSD* Post-Traumatic Stress Disorder, *ADHD* Attention Deficit Hyperactivity Disorder, *ED* eating disorder, *PHQ-9* patient health questionnaire-9, *GAD-7* generalized anxiety disorder scale-7, *SAS* zung self-rating anxiety scale, *OR* odds ratio, *IRR* incidence risk ratio

★: lockdown lasting less than one month ★★: lockdown lasting 1–3 months ★★★: lockdown lasting more than three months

### Poor mental health outcomes and risk factors

**Fig. 2:**
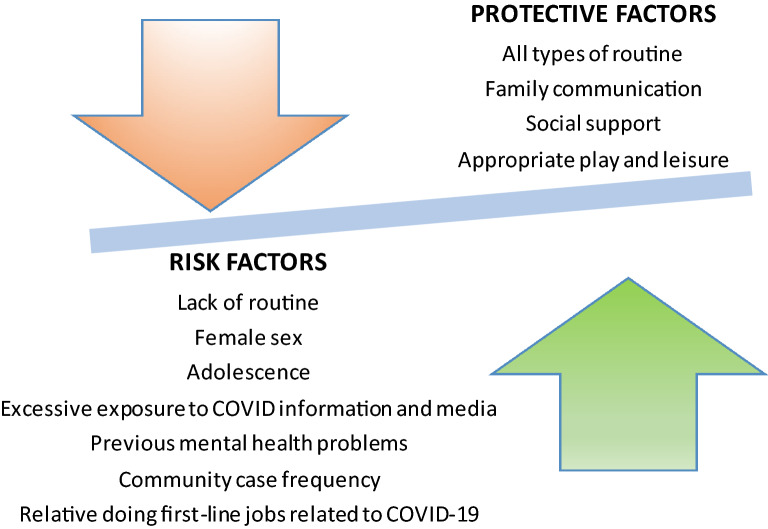
Risk and protective factors for anxiety symptoms/ affective symptoms in children and adolescents. This diagram refers to the risk and protective factors that are mentioned more than once within included studies

Most commonly evaluated outcomes in the included studies (see eTables 3–4) were anxiety (n = 35, 57.4%) and depression (*n* = 24, 39.3%). Symptoms of anxiety exacerbation were reported during or related/associated to lockdown in 57.4% of studies [1, 3, 5, 6, 11, 12, 16, 18, 20, 27, 39, 41, 47, 48, 51, 53, 56, 62, 72, 75, 79, 80, 82, 86–90, 94, 103, 107, 109, 110, 112]. The prevalence of symptoms of anxiety ranged from 1.8% [110] to 49.5% [41] between studies. 59.6% of young people reported increased ruminations [11]. 13.4% of children were found to be experiencing severe anxiety [109]. Meanwhile, 3.2% of children and adolescents met DSM-5 criteria for PTSD [110]. Risk factors to anxiety included lack of routine (*p* < 0.001) [6], female sex (*p* < 0.001) [20] (*p* = 0.041) [62], adolescence (*p* = 0.005) [44], excessive COVID-19 information (*p* < 0.05) defined by repeated exposure to COVID-19 related information [110], media exposure (OR = 2.4) [51], and being previously referred for psychiatric treatment (OR = 4.4) [51] (Fig. 2). An increased social media usage was associated with a higher risk of developing anxiety symptoms or and depression symptoms (OR = 1.83, *p* = 0.001) [89]. Children with Autism Spectrum Disorders (ASD) showed more anxiety than children without ASD during the lockdown [6].

Symptoms of depression were the second most commonly reported outcomes (*n* = 24, 39.3%) [2, 7, 12, 18, 20, 25, 41, 47, 48, 56, 62, 63, 72, 80, 83, 86, 89, 94, 106, 107, 109–112]. The prevalence of symptoms of depression ranged between 2.2% [110] and 63.8% [41] amongst studies. 7% of young people reported anhedonia [78]. The prevalence of children and adolescents with severe depression increased from 10 to 27% [41]. The prevalence of non-suicidal self-injury (OR = 1.35, *p* < 0.001), suicide ideation (OR = 1.32, *p* = 0.008), suicide planning (OR = 1.71, *p* < 0.001), and suicide attempts (OR = 1.74, *p* < 0.001) increased from November 2019 to May 2020 after lockdown [111]. Common risk factors for depression included female sex (*p* < 0.001) [18, 25, 62], being an adolescent (*p* < 0.01) [18, 83], a high amount of COVID-19 cases in the area (OR = 2.3, *p* < 0.001) [83, 107], and being exposed to a relative doing first-line job responsibilities related to COVID-19 (*p* < 0.05) [20]. Anger and irritability were common outcomes within children and adolescents ranging from 30.0% [78] to 51.3% [86] and from 16.7% [6] to 73.2% [86], respectively.

Symptoms of ADHD were frequently reported (*n* = 12, 19.7%) [17, 25, 34, 42, 59, 64, 74, 75, 85, 88, 91, 100]. Particularly, difficulties concentrating ranged from 55.9% [25] to 76.6% (*p* < 0.001) [75] in children and adolescents exposed to lockdown. Hyperactivity/inattention difficulties increased during lockdown (*p* < 0.001) [34, 42]. Exacerbation in symptoms of ADHD were related to increases in activity levels (50.1%), irritability (45.8%), disruptive behaviour (47.7%) [88], and conduct problems [74]. Risk factors for symptoms of ADHD worsening included sleep problems [17], being male (*p* < 0.001) [64], being a child compared to being an adolescent (*p* < 0.05) [85, 100], and parental stress (*p* < 0.001) [91].

Sleep disturbances were reported in a portion of the included studies (*n* = 11, 18.0%). 20% of children [79] and 55.6% of adolescents reported difficulty sleeping [25]. The proportion of children with sleep disorders increased from 40 to 62% during lockdown [54]. Young people showed difficulties initiating and maintaining sleep, the frequency of parasomnia increased [54]. Most studies reporting on sleep disturbances found that young people slept for longer during lockdown (*p* < 0.001) [39, 54]. Children went to bed ~ 53 min later (*p* < 0.0001) and woke up ~ 66 min later (*p* < 0.0001) than before the lockdown [34].

Longitudinal research findings showed a rise in children’s depressive symptoms [12] and anxiety symptoms compared to before the lockdown (*p* < 0.001) [19, 62]. Their risk increased when spending more time on COVID-19 media reports (*p* < 0.05) [110]. Furthermore, 41% of children and adolescents experienced a reactivation in eating disorder symptoms post lockdown, with a more pronounced reactivation of disordered eating seen in adolescents [44].

In lockdowns that lasted one month, previous service contact helped to alleviate anxiety [1]. However, longitudinal research findings showed that in a lockdown that lasted three months, children exhibited a deterioration in mental health symptoms, as reported by their parents, with a 10% increase in emotional symptoms, a 20% increase in hyperactivity/inattention, and a 35% increase in conduct problems [100]. Children and adolescents with special educational needs and neurodevelopmental disorders (NDD) showed more emotional symptoms, conduct problems, and hyperactivity/inattention scores than those without special educational needs and neurodevelopmental disorders [100]. Young people with NDD (28%), specifically ADHD, showed more conduct problems through lockdown, in comparison to neurotypicals controls (9%, *p* < 0.01) [74]. A decrease in therapy and rehabilitation support predicted externalising behaviours in children with NDDs [11].

### Good mental health and protective factors

31.4% of children, especially 9-year-olds (16.8%), were seen to be calmer during the pandemic than before it, and most children were able to cope and adapt to the lockdown measures (92.6%) [79]. Family relationships improved in 41.6% of households during lockdown [44]. Some children felt safe, relaxed, and happy when with their families [47, 48]. Healthy parent–child relationships were associated with positive parent–child communication [94]. Parents praised their children 67.6% more and spent 72.9% more time with them during the lockdown [88]; 58% of children were happy to spend more time with their families [89].

Some studies identified protective factors for mental health difficulties during the COVID-19 lockdown. Routines were associated with fewer symptoms of depression and improved mental health conditions in adolescents (*p* < 0.01) [38, 83]. Parent–child discussion was seen to mediate some anxiety (OR = − 1.6, *p* < 0.001) and depression (OR = − 1.9, *p* < 0.001) symptoms [94]. Parent–child discussion frequency was positively correlated to current life satisfaction (*p* < 0.05) [94]. A further protective factor for the mental health of children was play [47] (Fig. 2). Physical activity in children was associated with a lower hyperactivity-inattention risk (OR = 0.44, for 1–2 days activity a week; OR = 0.56, for < 2 days of activity a week) [59].

### Quality appraisal

The quality appraisal of the 61 studies is summarised in eTable 5. The overall average stars achieved through the 61 included studies was 7.0 stars (range = 4–9), which is considered as moderate quality. The domain of selection scored an average of 4.2/5.0 stars. The domain of comparability scored an average of 0.5/2.0 stars. The domain of outcome scored an average of 2.3/3.0 stars.

## Discussion

To our knowledge, this is the first systematic review to evaluate the effect of the COVID-19 lockdown on the mental health of children and adolescents. We found anxiety and depression to be the most common outcomes. A significant, substantial increase in depression and anxiety symptoms was seen in children during the lockdown compared to rates observed before the lockdown [12, 19, 62]. Other outcomes that seem to be associated with the COVID-19 lockdown are loneliness, psychological distress, anger, irritability, boredom, fear, and stress. Our results expand previous knowledge by identifying groups that may be at risk for mental health deterioration [6, 18, 20, 44, 48, 51, 62, 74, 83, 90, 100, 112]. During the lockdown, new psychiatric conditions may appear, while children and adolescents with previous mental health conditions, such as eating disorders, may experience a reactivation [44, 51].

The prevalence of PTSD seen in children exposed to COVID-19 was 3.2% [110]. This prevalence is lower than the one previously found in children quarantined or isolated due to the influenza A (H1N1) pandemic [92]. However, PTSD symptoms usually appear months after the traumatic experience, so it may be too early to estimate its scope at the moment. Furthermore, mental health in epidemics was more impaired in the phase following the acute outbreak, than in the initial phase [22]. Future research should evaluate a potential increase in PTSD symptoms and establish appropriate measures accordingly. Specifically, preventive measures in individuals at risk are recommended to avoid reaching these dramatically high rates observed in other health-related disasters. Teacher-based, resilience-focused interventions post-trauma have shown promising results [105]. Furthermore, meta-analytical evidence suggests trauma-focused psychotherapy might be effective for the prevention of PTSD in patients with acute stress symptoms [93].

Individuals with previous eating disorders have been among the most intensively affected. 41% of young people under clinical care experienced a reactivation in eating disorder symptoms post lockdown [44], particularly those with low self-directedness and less adaptive coping strategies [9]. Lack of weight monitoring during confinement may have played a role here [9]. Individuals suffering from eating disorders struggled to maintain feeding routines and research shows COVID-19 lockdown to significantly correlate with symptoms of disordered eating [61]. Considering eating disorders have the highest mortality rate [102], there should be an increased utilisation of digital tools to support those with eating disorders in the context of COVID-19 [28].

This review found sociodemographic characteristics influencing the development of poor mental health outcomes associated with COVID-19 lockdown to include older age (13–15 vs. 6–12, *p* < 0.03 [18, 83]) and female sex [18, 20, 62, 90]. Adolescents have been previously identified as a vulnerable group, going through an important period in their development [15] where peer relationships are of the most importance. Older adolescents displayed more depressive symptoms than younger adolescents during the lockdown [18]. This may be because the onset of depression increases as children transition into adolescence [68]. A further explanation is that adolescents are in particular need of social contact and interpersonal relationships. The period of adolescence is a motivator for peer connection [36] and the desire for peer and social support [37], which aids the development of identity [67]. However, during the lockdown, they need to attend online learning, cope with school closures and adapt to a mandatory decrease in social relations [60].

Another vulnerable group identified by this systematic review are the children and adolescents and with previous mental health difficulties or with “special educational needs and disabilities” (SEND) and/or neurodevelopmental disorders [6, 44, 51, 100]. One of the reasons children and adolescents with neurodevelopmental disorders are highly vulnerable to suffering psychological distress is that while they usually prefer routine and predictable environments, the COVID-19 pandemic is a situation of fast-paced changes [24]. Children and adolescents with SEND, ASD and/or disabilities have had their carefully constructed routines suddenly disrupted [98] alongside affected support networks resulting in a higher risk of experiencing poor mental health and increased stress during the unprecedented lockdown [8]. With special education closed, these young people may struggle more with adapting to virtual schooling. Social factors in these children and adolescents are also important. 24% of teachers claimed families of those with SEND and/or disabilities don’t have access to sufficiently powerful devices or software required to download or access digital materials required [77], which further complicates their situation. As a result of lockdown, symptoms of ADHD were seen to worsen [25, 34, 42, 59, 75, 85, 88, 100]. Certain home environments (e.g., having a garden or adequate space at home) had a positive impact on ADHD symptoms. However, limited academic adjustments for children with ADHD were reported by parents, resulting in difficulties to carry out school-related tasks [13].

Identifying risk and protective factors is crucial for clinical practice to identify individuals who are more vulnerable to poor mental health outcomes and to develop clinical practices and public health strategies to reduce the negative impact of lockdown on children and adolescents. Risk factors include lack of routine [83], the form of internet usage [19], COVID-19 media exposure, and a relative doing first-line job responsibilities related to COVID-19 [20]. Quarantine affects the structure of children and adolescents days’ [46]. Therefore, schools play an important role over lockdown as they’re able to provide structure into young peoples’ days which is seen to be protective, as long as they don’t overburden young people [101].

In addition, school closure has been identified as a key stressor for some young people [7]. Significant associations have been found between emotional reactions and home-school experiences [53]. 56% of those experiencing psychosocial problems as a result of lockdown reported that this was related to not being able to attend school [89]. Furthermore, during school closure, child protection referrals from schools have decreased compared to previous years [99]. A decrease in help-seeking behaviour and access to care may have contributed negatively to the mental health of children and adolescents. The impact of school closure has not been equal for all. Children in the primary school reported fewer depressive symptoms compared to children in middle school [106].

Internet usage reduces the time being spent doing other beneficial activities and may adversely affect children’s emotional health and psychological wellbeing [66]. Problematic internet usage was seen to result in psychological distress characterised by excessive time spent gaming, on one’s smartphone, and on social media [19]. This has been supported by research finding that problematic internet use is associated with depression, anxiety, and other health problems [35]. Excessive time spent on the internet may occur as children are bored at home, isolated from peers, and cannot attend regular extracurricular activities. Research has found those in social isolation to have a higher level of media contact, with more exposure to COVID-19 related information [58]. During pandemics and epidemics, media exposure is reported to worsen severe mental health outcomes [21]. For instance, excessive COVID-19 media exposure has been associated with an increase in anxiety levels and stress [40]. It would be recommendable for parents to limit the time children and adolescents spend using the internet and to model positive coping behaviours [97] to reduce stress, encouraging children to carry out other activities, for example, listening to music [43], reading together, and playing board games together [55]. Physical activity also reduced hyperactivity-inattention risk in children [59], which could be encouraged or recommended by caregivers. Parents and health professionals should also make sure children and adolescents get only truthful and balanced information. These aspects are a real challenge for parents that need to work remotely and simultaneously take care of their children.

Previous research has shown that family environment, parental practices, and methods of coping affect children’s post-disaster mental health [23]. However, the lockdown has not negatively impacted everyone and may have been beneficial for some relationships to develop. Parent–child discussion was seen to be protective against child mental health, specifically anxiety, depression and stress, and is related to life satisfaction [94]. Perceived family relationship improvements may be a consequence of families being able to spend more quality time with one another due to remote working [104]; however, this can result in mental strain on some parents, especially parents of children with SEND [33]. Family relationships may serve to support child adjustment when faced with adversity [26]. In addition to this, experiencing collective family major life challenges may promote positive family transformations [65].

Numerous changes in mental health provision have occurred since the start of the COVID-19 pandemic lockdown to minimise the infection rate, such as a rise in community support services and implementing inpatient infection-control measures. To ensure continuity of mental healthcare for service users, mental health services have had to adapt mainly via adopting more telehealth methods [71, 84]. The COVID-19 lockdown has resulted in a rise in virtual, remote therapy, which may have future implications for service provision after COVID-19. For example, telehealth will allow those who live in remote areas to access mental healthcare more easily. Since COVID-19, telemedicine has been expanded, so more people are eligible for it and rules have been relaxed for health insurance providers and doctors. Telehealth reduces barriers to access, is more cost-effective, and has a wide availability of services within paediatric care [81]. However, telemedicine is limited by one’s technology literacy, psychological resistance to new methods, and cultural background [52].

The findings highlighted in the present work have further clinical implications. Governments should ensure that lockdowns be as short as possible to limit the psychological effects of lockdown on children and adolescents, while protecting their safety. Governments should also aim to release COVID-19 information, information about prevention measures, and lockdown updates while ensuring that the information provided is accurate [95]. The public should have access to age-appropriate resources such as improving sleep hygiene, maintaining a balanced diet, routine keeping, and mental healthcare [101] to educate young people on keeping healthy to prevent negative psychological effects. Sleep quality [17, 25, 34, 39, 72, 79, 86] and sleep disorders [10, 54, 109] were seen to worsen during the pandemic. Therefore, easily accessible sleep hygiene resources for children may be protective against adverse sleep effects. Moreover, an increase in funding allocation to mental health services needs to be provided along with trained staff to facilitate care and ensure continuity of care for vulnerable populations and cope with the long-term mental health effects the COVID-19 lockdown may have.

Another fundamental clinical implication highlighted by this review is that the break of care due to the lockdown [45] may have delayed access to treatment, pushing the course of recovery back [84]. Clinicians should follow up on those who have experienced a break of care as they may be more vulnerable to reactivation of symptoms post lockdown. This can be done by having regular mental health check-ups for vulnerable groups to assess their mental state. Unfortunately, a reduction in self-help-seeking behaviours has been observed. For instance, hospital presentations for self-harm decreased in 2020, compared to 2019 [76] regardless of an increase in these behaviours seen during the lockdown [111]. Attention should be paid to the more vulnerable groups post-lockdown when it comes to accessing mental health care and parents should also be provided psychoeducational resources to help identify psychological distress in their children [101].

We need to balance health and safety on one side and mental health and normal psychological development on the other. Short lockdowns seem to be better tolerated, especially with previous service contact [1]. However, the longer the lockdown lasts the more support children may need. For instance, children under lockdown for three months exhibited far more conduct problems [100]. Lockdowns should be made as short as possible and should assess the benefit/risk balance when deciding how long lockdowns should last to limit mental health consequences.

This study has some limitations that must be considered. First, this review does not meta-analytically evaluate the magnitude and consistency of the mental health outcomes described due to the heterogeneity of the outcomes and measurement methods. Second, evidence on the effect of lockdown on low-income households and low-income countries was limited. Further research is needed to draw conclusions on whether the impact of COVID-19 lockdown on the mental health of children and adolescents is different or not between low- and high-income countries. A third limitation is that some studies (62.3%) did not provide details about the duration of the lockdown established, precluding drawing further conclusions from our end.

Fourth, most studies included (73.8%) were cross-sectional, limiting causal inference. The inclusion of some cohort studies evaluating children and adolescents before and after the lockdown, allowed to evaluate more precisely the effect of lockdown while helped control some situational confounders. A fifth limitation would be that most included studies focussed on psychological reactions and symptomatology rather than the appearance of mental disorders, which has implications for practice. Future longitudinal studies should follow children and adolescents who experienced poor mental health during lockdown to see if they recover, mental health difficulties persist, or they crystalise into full-blown mental disorders. Lastly, most studies were conducted online, where it would be difficult for children to ask for clarification around the questions they did not understand. Due to the lockdown, children and adolescents could not attend research centres for their safety.

## Conclusions

The COVID-19 lockdown has resulted in psychological distress and highlighted vulnerable groups such as those with mental health difficulties, and risk factors such as lack of routine and excessive COVID-19 media exposure. However, for some families being able to spend more quality time together has been positive. Supporting the mental health needs of children and adolescents at risk is key. Clinical guidelines to alleviate the negative effects of COVID-19 lockdown and public health strategies to support this population need to be developed.

## Supplementary Information

Below is the link to the electronic supplementary material.

Supplementary file1 (DOCX 172 KB)
