# Supplementary material for: The impact of COVID-19 lockdown on child and adolescent mental health: systematic review
Source: Eur Child Adolesc Psychiatry. 2021 Aug 18;32(7):1151–77. doi: 10.1007/s00787-021-01856-w (PMC8371430; doi:10.1007/s00787-021-01856-w)
Supplement: Supplementary file 1 — Supplementary file1 (DOCX 172 KB) [file 787_2021_1856_MOESM1_ESM.docx]

**SUPPLEMENTARY MATERIAL**

**eTable 1:** PRISMA statement and checklist.…..…..…..…..…..…..…..…..…..…..…..…..…..….…..…..…..…..…..…....……...pages 2,3

**eTable 2:** Lockdown definitions and length…………………………………………….………………………..………..….…….pages 4,5,6

**eTable 3:** Summary of instruments used across studies to assess outcomes.…..…..…..…..…..….……..…..…..…..…...…..pages 7,8

**eTable 4:** Instruments and reports………………………………………………………………………………………………..… pages 9-11

**eTable 5:** Quality assessment results……………………………………………………………………………………………...pages 12-15

**eMethods 1:** Literature search terms……………………………………………………………………………………………………page 16

**eMethods 2:** Newcastle-Ottawa Scale (NOS) adapted for cross-sectional studies...…..…...…..…..…..…...…………..…pages 17-19

**This supplementary material has been provided by the authors to give readers additional information about their work.**

**eTable 1: PRISMA statement and checklist**

| **Section/topic** | **#** | **Checklist item** | **Page** |
| --- | --- | --- | --- |
| **TITLE** | | |  |
| Title | 1 | Identify the report as a systematic review, meta-analysis, or both. | 1 |
| **ABSTRACT** | | |  |
| Structured summary | 2 | Provide a structured summary including, as applicable: background; objectives; data sources; study eligibility criteria, participants, and interventions; study appraisal and synthesis methods; results; limitations; conclusions and implications of key findings; systematic review registration number. | 2 |
| **INTRODUCTION** | | |  |
| Rationale | 3 | Describe the rationale for the review in the context of what is already known. | 3 |
| Objectives | 4 | Provide an explicit statement of questions being addressed with reference to participants, interventions, comparisons, outcomes, and study design (PICOS). | 3 |
| **METHODS** | | | |
| Protocol and registration | 5 | Indicate if a review protocol exists, if and where it can be accessed (e.g., Web address), and, if available, provide registration information including registration number. | 3 |
| Eligibility criteria | 6 | Specify study characteristics (e.g., PICOS, length of follow-up) and report characteristics (e.g., years considered, language, publication status) used as criteria for eligibility, giving rationale. | 3/4 |
| Information sources | 7 | Describe all information sources (e.g., databases with dates of coverage, contact with study authors to identify additional studies) in the search and date last searched. | 3 |
| Search | 8 | Present full electronic search strategy for at least one database, including any limits used, such that it could be repeated. | S16 |
| Study selection | 9 | State the process for selecting studies (i.e., screening, eligibility, included in systematic review, and, if applicable, included in the meta-analysis). | 3/4 |
| Data collection process | 10 | Describe method of data extraction from reports (e.g., piloted forms, independently, in duplicate) and any processes for obtaining and confirming data from investigators. | 4 |
| Data items | 11 | List and define all variables for which data were sought (e.g., PICOS, funding sources) and any assumptions and simplifications made. | 4 |
| Risk of bias in individual studies | 12 | Describe methods used for assessing risk of bias of individual studies (including specification of whether this was done at the study or outcome level), and how this information is to be used in any data synthesis. | 4 |
| Summary measures | 13 | State the principal summary measures. | D.n.a. |
| Risk of bias across studies | 15 | Specify any assessment of risk of bias (i.e. Newcastle-Ottawa Scale (NOS), that may affect the cumulative evidence. | 4 |
| Additional analyses | 16 | Describe methods of additional analyses (e.g., sensitivity or subgroup analyses, meta-regression), if done, indicating which were pre-specified. | D.n.a. |
| **RESULTS**   | | | |
| Study selection | 17 | Give numbers of studies screened, assessed for eligibility, and included in the review, with reasons for exclusions at each stage, ideally with a flow diagram. | 4, 13 |
| Study characteristics | 18 | For each study, present characteristics for which data were extracted (e.g., study size, PICOS, follow-up period) and provide the citations. | 15-19 |
| Risk of bias within studies | 19 | Present data on risk of bias of each study and, if available, any outcome level assessment (see item 12). | S12-15 |
| Results of individual studies | 20 | For all outcomes considered (benefits or harms), present, for each study a summary data for each intervention group | 14-18 |
| Synthesis of results | 21 | Present results of study analyzed. | 4-7 |
| Risk of bias across studies | 22 | Present results of any assessment of risk of bias across studies (see Item 15). | 7 |
| Additional analysis | 23 | Give results of additional analyses, if done (e.g., sensitivity or subgroup analyses, meta-regression [see Item 16]). | N.a. |
| **DISCUSSION**   | | | |
| Summary of evidence | 24 | Summarize the main findings including the strength of evidence for each main outcome; consider their relevance to key groups (e.g., healthcare providers, users, and policy makers). | 7-10 |
| Limitations | 25 | Discuss limitations at study and outcome level (e.g., risk of bias), and at review-level (e.g., incomplete retrieval of identified research, reporting bias). | 11 |
| Conclusions | 26 | Provide a general interpretation of the results in the context of other evidence, and implications for future research. | 11 |
| **FUNDING**   | | | |
| Funding | 27 | Describe sources of funding for the systematic review and other support (e.g., supply of data); role of funders for the systematic review. | 11 |

**eTable 2: Lockdown definitions and length**

| **Lead Author/ year** | **Lockdown detail** | **Length of lockdown** |
| --- | --- | --- |
| Abawi et al., 2020 [1] | Governmental lockdown measures | 1 month (April 2020) |
| Abdulah et al., 2020 [2] | Home confinement for at least a month during the COVID-19 outbreak | N.A. |
| Achterberg et al., 2020 [3] | COVID-19 lockdown | N.A. |
| Adibelli et al., 2020 [4] | COVID-19 lockdown | 1 month (March-April 2020) |
| Alves et al., 2020 [5] | Schools were closed | 1 month, 1 week (April 22^nd^ - May 29^th^ 2020) |
| Amorim et al., 2020 [6] | Home isolation during quarantine due to COVID-19 | N.A. |
| Asanov et al., 2021 [7] | School closure, national lockdown, and quarantine | Remainder of academic year (starting March 2020) |
| Baptista et al., 2020 [8] | School closures | Duration of study (starting March 2020) |
| Bentenuto et al., 2021 [9] | N.A. | N.A. |
| Bignardi et al., 2020 [10] | School closures and social distancing | 3 months (April-June 2020) |
| Cauberghe et al., 2020 [11] | Lockdown began on March 18, 2020, and included a closure of schools, restaurants and bars, leisure activities, and nonessential stores, and the borders. People were told to work from home, and were only allowed to have physical contact with members of the same household | N.A. |
| Cetin et al., 2020 [12] | School closures and individuals under 20 were required to confine at home | 1 month (April-May 2020) |
| Chen et al., 2020a [14] | School suspension | N.A. |
| Chen et al., 2020b [13] | School suspension with online learning and daily activities being conducted indoors | N.A. |
| Chen et al., 2020 [15] | Data collected during a complete lockdown between February 22, 2020, and March 8, 2020 | N.A. |
| Commodari et al., 2020 [16] | 31% of the students lived in “red zones” where there were the strictest containment measures. Lockdown | 1 month (March 9th - May 4th, 2020) |
| Conti et al., 2020 [17] | COVID-19 lockdown | 1 month |
| Cusinato et al., 2020 [18] | School closures | 50.6 ± 20.7 days |
| Di Giorgio et al., 2020 [19] | Social restrictions | 1 month (March-April 2020) |
| Ezpeleta et al., 2020 [20] | Lockdown spanned from the 13th of March 2020 up to the 24th of May 2020. The lockdown lasted for 72 days when non-essential services and schools were suspended, and people were told to stay at home | 72 days |
| Francisco et al., 2020 [21] | Quarantine | 1 month (March-April 2020) |
| Giannopoulou et al., 2021 [22] | School and university closures. Restriction of movement where people could only leave their houses for specific reasons and with a special permit | 1 month (March-April 2020) |
| Gimenez-Dasi et al., 2020 [23] | Confinement | 1.5 months |
| Graell et al., 2020 [24] | ED unit during the 8-week COVID-19 confinement period resulted in the cancellation of non-urgent face-to-face outpatient visits and day-hospital care for child and adolescent psychiatry | 8 weeks |
| Idoiaga et al., 2020a [25] | Home confinement from March 13th to April 26th, 2020. | 44 days (March 13^th^ - April 26^th,^ 2020) |
| Idoiaga et al., 2020b [26] | School suspension and home confinement since March 13, 2020 | 6 weeks |
| Kılınçel et al., 2020 [27] | School suspension and home quarantine | N.A. |
| Larsen et al., 2020 [28] | Public government induced lockdown | Over 1 month (1^st^ April-25^th^ May 2020) |
| Lecuelle et al., 2020 [29] | COVID-19 lockdown | 2 months |
| Liang et al., 2020 [30] | School and university closure | N.A. |
| Liebana-Presa et al., 2020 [31] | Home confinement | 1 month |
| Liu et al., 2020 [32] | School closure | N.A. |
| Magson et al., 2020 [33] | School suspension, online learning, and government restrictions | N.A. |
| Majeed et al., 2020 [34] | School closures | N.A. |
| Mallik et al., 2021 [35] | COVID-19 lockdown | N.A. |
| Morgul et al., 2020 [37] | School closure and restrictions around movements | 1 month |
| Mourouvaye et al., 2020 [38] | COVID-19 lockdown | 2 months (March-May 2020) |
| Nonweilier et al., 2020 [39] | COVID-19 lockdown | 3 months (April-June 2020) |
| Orgilés et al., 2020 [40] | School suspension and restrictions around public gathering | N.A. |
| Patra et al., 2020 [41] | Home quarantine and school suspension from 25th March 2020 | N.A. |
| Pisano et al., 2020 [42] | School suspension and home isolation announced on 5th March 2020 | N.A. |
| Pons et al., 2020 [43] | Home confinement | 72.7 ± 12.0, 50-100 days |
| Radwan et al., 2020 [44] | School closures | 1 month (July-August 2020) |
| Ren et al., 2020 [45] | School suspension and lockdown | 2 months |
| Romero et al., 2020 [46] | Home confinement, banning of all non-essential activity, school closure. | 43 days |
| Sama et al., 2020 [47] | 42-day nationwide lockdown | 42 days (25^th^ March-3^rd^ May 2020) |
| Saurabh et al., 2020 [48] | Lockdown from the 25th of March, 2020 | N.A. |
| Shah et al., 2020a [50] | Home confinement, online learning, school closures | N.A. |
| Shah et al., 2020b[49] | School suspension and online learning | N.A. |
| Smirni et al., 2020 [51] | Home isolation from 15th April to 15th May, 2020 | N.A. |
| Spinelli et al., 2020 [52] | National restrictions | 2 months (April-May 2020) |
| Tang et al., 2020 [53] | Lockdown from the 24th of January 2020 until the 23rd of March 2020 led to the closure of public venues, cancellation of public events and home isolation. Lockdown was stricter in high infection areas | Over 1 month (2^nd^ March-27^th^ April 2020) |
| Troncone et al., 2020 [54] | Travel restrictions, social isolation, school closures, suspension of non-essential services | 2 months (March-May 2020) |
| Waite et al., 2020 [55] | Surveys conducted between March and May 2020 when the UK was in lockdown | 3 months (March-May 2020) |
| Wiguna et al., 2020 [56] | Students advised to stay and study from home | 2 months |
| Xiang et al., 2020 [57] | School closures | 3 months (January-March 2020) |
| Xie et al., 2020 [58] | School suspension and home isolation | (2 and a half months (2^nd^ March-27^th^ April 2020) |
| Yeasmin et al., 2020 [59] | School suspension starting from the 26th of March, 2020 | N.A. |
| Yue et al., 2020 [60] | All children and their parents were staying at home with the quarantine order | N.A. |
| Zhang et al., 2020 [61] | School closures | 3 months |
| Zijlmans et al., 2020 [62] | Lockdown began on the 15^th^ of March 2020. School, sport and leisure facilities, bar, and restaurant closures. Adults worked from home. People are still allowed outside, to have 3 guests over, and children under 12 could play with friends outside. | 2 months (April-May 2020) |

**eTable 3: Summary of instruments used across studies to assess outcomes.**

| **Assessment** | **Instruments** |
| --- | --- |
| Qualitative | Semi-structured interviews, structured interviews alongside arts-based methods and free association tasks |
| Quality of life | Pediatric Quality of Life Inventory (PedsQL), Kid-KINDL |
| Child perception of parent rearing | Short Egna Minnen Beträffande Uppfostran (S-EMBU) |
| COVID-19 measures | Risk Perception of Infectious Diseases Questionnaire, Impact Scale of the COVID-19 and home confinement on children and adolescents |
| Substance Use | Cannabis Use: Intention Questionnaire (CUIQ) |
| General health | 24-hour Physical Activity Recall (PAR), Holistic Monitoring Questionnaire (HMQ) |
| Parent based measures | Parent Stress Scale (PSS), Coparenting Relationship Scale (CRS), Strengths and Difficulties Questionnaire - Parent version (SDQ-P), Parenting-Stress Index Short Form (PSI), Family daily routines and children’s emotional and behavioural symptoms questionnaire |
| General mental health, coping and behavioural outcomes | Strengths and Difficulties Questionnaire (SDQ), the Brief-COPE scale, the Child Routines Inventory (CRI), the Student Life Satisfaction Scale (SLSS), the Life Satisfaction assessment (SWLS), the Short Mood and Feelings Questionnaire - Child version (SMFQ-C), World Mental Health Barriers to Service Scale, Emotional Awareness Questionnaire (EAQ), and the Child Behaviour Checklist (CBCL), Positive and Negative Affect Schedule for Children (PANAS-C), Mental Health Inventory (MHI-5), Behavior Rating Inventory of Executive Function - preschool version (BRIEF-P), Difficulties in Emotional Regulation (DERS), System of Evaluation of Children and Adolescents (SENA), Trait Meta-Mood Scale (TMMS), General Health Questionnaire-12 (GHQ-12), Student Stress Inventory-Stress Manifestations (SSI-SM), Kessler Psychological Distress Scale (K6) |
| Symptoms of depression | Centre of Epidemiological Studies Depression Scale (CES-DC), Birleson Depression Self-Rating Scale for Children (DSRS-C), and Child Depression Inventory Short Form (CDI-S) |
| Symptoms of anxiety | General Anxiety Disorder Scale (GAD-7/GAD-6), Screen for Child Anxiety Related Disorders (SCARED), State Trait Anxiety Inventory (STAI), State-Trait Anxiety Inventory for Children (STAIC), Spence’s Children’s Anxiety Scale for Children (SCAS-C) and for Parents (SCAS-P), and Zung Self-rating Anxiety Scale (SAS) |
| Symptoms of anxiety and depression | The Depression, Anxiety, Stress Scale (DASS-21), Revised Child Anxiety and Depression Scale (RCADS) and the Self-Rating Depression Scale (SDS), Patient Health Questionnaire for Depression and Anxiety (PHQ-4) |
| Symptoms of PTSD | PTSD Checklist for DSM-5 (PCL-5), Children’s Impact of Event Scale (CRIES-8) |
| Sleep | Children’s Sleep Habits Questionnaire (CSHQ), Children’s Chronotype Questionnaire (CCQ), The Pittsburgh Sleep Quality Index (PSQI), Subjective Time Questionnaire (STQ), Sleep Disorder Scale for Children (SDSC) |
| Loneliness | Revised UCLA Loneliness Scale (RULS-6) and the UCLA Loneliness Scale |
| ADHD | Swanson Nolan Pelham (SNAP-IV) |
| Resilience | Connor-Davidson Resilience Scale (CD-RISC-10) |
| Eating | Eating Attitudes Test-26 (EAT-26), Children Eating Attitudes Test (ChEAT) |
| Technology-based addictions | Smartphone Application-Based Addiction Scale (SABAS), Bergen Social Media Addiction Scale (BSMAS), and Internet Gaming Disorder Scale - Short Form (IGDS9-SF) |
| Outcomes | Patient-Reported Outcome Measures (PROM), Brief Symptom Inventory (BSI), Pediatric Symptom Checklist (PSC), and Ad-Hoc Questionnaire |

**eTable IV: Instruments and reports**

| **Lead Author/ year** | **Instruments** | **Report** |
| --- | --- | --- |
| Abawi et al., 2020 [1] | PedsQL and semi-structured telephone interview. | Parent and child report |
| Abdulah et al., 2020 [2] | Arts based measures - drawing method. | Child report |
| Achterberg et al., 2020 [3] | Ad Hoc Questionnaire, BSI | Parent and child report |
| Adibelli et al., 2020 [4] | Kid-KINDL | Parent report |
| Alves et al., 2020[5] | PAR, STAIC, PANAS-C | Parent and child report |
| Amorim et al., 2020 [6] | Ad Hoc Questionnaire | Parent report |
| Asanov et al., 2021 [7] | MHI-5 | Child report |
| Baptista et al., 2020 [8] | Ad Hoc Questionnaire | Parent report |
| Bentenuto et al., 2021 [9] | PSS, CRS, SDQ | Parent report |
| Bignardi et al., 2020 [10] | SDQ and RCADS. | Parent and child report |
| Cauberghe et al., 2020 [11] | CES-DC, GAD-7, RULS-6, Brief-COPE | Child report |
| Cetin et al., 2020 [12] | CRIES-8, CSHQ, CCQ | Parent report |
| Chen et al., 2020a [14] | SABAS, BSMAS, IGDS, DASS-21 | Child report |
| Chen et al., 2020b[13] | DSRS-C, SCARED | Child report |
| Chen et al., 2020 [15] | S-EMBU, PHQ-9, GAD-7 | Child report |
| Commodari et al., 2020 [16] | Risk Perception of Infectious Diseases Questionnaire | Child report |
| Conti et al., 2020 [17] | CBCL | Parent report |
| Cusinato et al., 2020 [18] | Ad Hoc Questionnaire | Parent report |
| Di Giorgio et al., 2020 [19] | PSQI, STQ, BRIEF-P, SDQ-P. DERS | Parent report |
| Ezpeleta et al., 2020 [20] | SDQ | Child report |
| Francisco et al., 2020 [21] | Ad Hoc Questionnaire | Parent report |
| Giannopoulou et al., 2021 [22] | GAD-7, PHQ-9 | Child report |
| Gimenez-Dasi et al., 2020 [23] | SENA | Parent and child report |
| Graell et al., 2020 [24] | Combined teletherapy method and retrospective review of patient records | Child report |
| Idoiaga et al., 2020a [25] | Structured interview about child's lockdown activities, needs, and feelings. | Child report |
| Idoiaga et al., 2020b [26] | Interview and free association exercise | Child report |
| Kılınçel et al., 2020 [27] | STAI, UCLA loneliness scale | Child report |
| Larsen et al., 2010 [28] | SMFQ, SCARED, and interviews. | Child report |
| Lecuelle et al., 2020 [29] | SDSC | Parent report |
| Liang et al., 2020 [30] | Impact Scale of the COVID-19 and home confinement on children | Parent report |
| Liebana-Presa et al., 2020 [31] | TMMS, SSI-SM, CUIQ | Child report |
| Liu et al., 2020 [32] | SDQ | Parent and child report |
| Magson et al., 2020 [33] | SCAS-C, SMFQ-C, SLSS | Child report |
| Majeed et al., 2020 [34] | DSM | Child report |
| Mallik et al., 2021 [35] | SDQ | Parent report |
| Morgul et al., 2020 [37] | Family daily routines and child’s emotional and behavioural symptoms questionnaire, K6 | Parent report |
| Mourouvaye et al., 2020 [38] | N.A. | Child report |
| Nonweilier et al., 2020 [39] | SDQ | Parent report |
| Orgilés et al., 2020 [40] | Ad Hoc Questionnaire | Parent report |
| Patra et al., 2020 [41] | SNAP-IV, Semi-structured interview, WMH | Parent and child report |
| Pisano et al., 2020 [42] | Ad Hoc Questionnaire | Parent report |
| Pons et al., 2020 [43] | HMQ, GHQ-12 | Child report |
| Radwan et al., 2020 [44] | Ad Hoc Questionnaire | Child report |
| Ren et al., 2020 [45] | CES-DC, adapted CRI, Ad Hoc Questionnaire | Child report |
| Romero et al., 2020 [46] | CD-RISC-10, PHQ-4, SDQ | Parent report |
| Sama et al., 2020 [47] | Ad Hoc Questionnaire | Parent report |
| Saurabh et al., 2020 [48] | Structured interview regarding their compliance and psychological distress | Parent and child report |
| Shah et al., 2020a [50] | PSC | Child report |
| Shah et al., 2020b [49] | Ad Hoc Questionnaire | Parent report |
| Smirni et al., 2020 [51] | SAS, EAQ | Child report |
| Spinelli et al., 2020 [52] | PSI, DASS, SDQ | Parent report |
| Tang et al., 2020 [53] | DASS-21, SWLS, Ad Hoc questionnaire, Parent-Child interview | Parent and child report |
| Troncone et al., 2020 [54] | EAT-26, ChEAT | Child report |
| Waite et al., 2020 [55] | SDQ | Parent report |
| Wiguna et al., 2020 [56] | SDQ | Child report |
| Xiang et al., 2020 [57] | CDI-S | Parent and child report |
| Xie et al., 2020 [58] | CDI-S, SCARED | Child report |
| Yeasmin et al., 2020 [59] | RCADS, GAD-6, SCAS-P, CBCL | Parent report |
| Yue et al., 2020 [60] | SAS, CES-DC, SDS, PCL-5. | Parent and child report |
| Zhang et al., 2020 [61] | Ad Hoc Questionnaire | Child report |
| Zijlmans et al., 2020 [62] | Ad Hoc Questionnaire and PROM | Parent and child report |

PedsQL: Pediatric Quality of Life Inventory; CES-DC: Centre of Epidemiological Studies Depression Scale; GAD-7: Generalised Anxiety Disorder Scale; RULS-6: Revised UCLA Loneliness Scale; Brief-COPE: Brief-Coping Scale; SABAS: Smartphone Application−Based Addiction Scale; BSMAS: Bergen Social Media Addiction Scale; IGCS-SF9: Internet Gaming Disorder Scale−Short Form; DASS-21: The Depression, Anxiety, Stress Scale−21; DSRS-C: Depression Self-Rating Scale for Children; SCARED: Screen for Child Anxiety Related Disorders; S-EMBU: Short Egna Minnen Beträffande Uppfostran; PHQ-9: Patient Health Questionnaire-9; SDQ: Strengths and Difficulties Questionnaire; STAI: State-Trait Anxiety Inventory; UCLA loneliness scale; SCAS-C: Spence Children’s Anxiety Scale; SMFQ-C: Short Mood and Feelings Questionnaire—Child Version; SLSS: Student's Life Satisfaction Scale; SNAP-IV: Swanson Nolan Pelham; WMH: World Mental Health Survey Barriers to Service Scale; CRI: Child Routines Inventory; SAS: Zung Self-Rating Anxiety Scale; EAQ: Italian Emotion Awareness Questionnaire; DASS-21: Depression, anxiety, and stress scale; SWLS: Satisfaction With Life Scale; CDI-S: Children’s Depression Inventory–Short Form; CBCL: Child Behavior Checklist; GAD-6: Generalized Anxiety Disorder; RCADS: Revised Child Anxiety and Depression Scale; SCAS-P: Spence Child Anxiety Scale for Parents; PROM: Patient-Reported Outcome Measures; SDS: Self-Rating Depression Scale; PCL-5: PTSD Checklist for DSM-5; BSI: Brief Symptom Inventory; SENA: System of Evaluation of Children and Adolescents; SSI-SM: Student Stress Inventory-Stress Manifestations; CUIQ: Cannabis Use: Intention Questionnaire; HMQ: Holistic Monitoring Questionnaire; GHQ-12: General Health Questionnaire-12; CD-RISC-10: Connor-Davidson Resilience Scale; PHQ-4: Pediatric Health Questionnaire for Depression and Anxiety; K6: Kessler Psychological Distress Scale; PSC: Pediatric symptom checklist - youth short version; PSI: Parenting-Stress Index - Short Form; EAT-26: Eating Attitudes Test-26; ChEAT: Children Eating Attitudes Test; Sleep Disorder Scale for Children (SDSC); Trait Meta-Mood Scale (TMMS)

**eTable 5:** Quality assessment results based off the Newcastle-Ottawa Scale (NOS) adapted for cross-sectional studies

| Study | Design | Sample representativeness | Sample size | Non-response rate | Exposure definition | Adjustment for confounders | Assessment of the outcome | Statistical test | Score (0-10) |
| --- | --- | --- | --- | --- | --- | --- | --- | --- | --- |
| Abawi et al., 2020. [1] | Cross sectional | 1 | 1 | 1 | 2 | 0 | 2 | 1 | 8 |
| Abdulah et al., 2020 [2] | Cross sectional | 0 | 0 | 1 | 2 | 0 | 1 | 2 | 6 |
| Achterberg et al., 2020 [3] | Longitudinal | 1 | 1 | 0 | 2 | 0 | 0 | 1 | 5 |
| Adibelli et al., 2020 [4] | Cross sectional | 1 | 1 | 0 | 2 | 0 | 2 | 1 | 7 |
| Alves et al., 2020 [5] | Longitudinal | 1 | 1 | 1 | 2 | 0 | 2 | 1 | 8 |
| Amorim et al., 2020 [6] | Cross sectional | 0 | 1 | 1 | 2 | 2 | 1 | 1 | 8 |
| Asanov et al., 2021 [7] | Cross sectional | 1 | 1 | 1 | 2 | 0 | 1 | 1 | 7 |
| Baptista et al., 2020 [8] | Cross sectional | 1 | 1 | 1 | 2 | 0 | 1 | 1 | 7 |
| Bentenuto et al., 2021 [9] | Cross sectional | 1 | 1 | 0 | 2 | 2 | 2 | 1 | 9 |
| Bignardi et al., 2020 [10] | Longitudinal | 0 | 1 | 0 | 2 | 1 | 1 | 1 | 6 |
| Cauberghe et al., 2020 [11] | Cross sectional | 0 | 1 | 0 | 2 | 0 | 1 | 1 | 5 |
| Cetin et al., 2020 [12] | Cross sectional | 1 | 1 | 1 | 2 | 0 | 2 | 1 | 8 |
| Chen et al., 2020a [14] | Longitudinal | 1 | 1 | 1 | 2 | 1 | 2 | 1 | 9 |
| Chen et al., 2020b [13] | Cross sectional | 1 | 1 | 1 | 2 | 0 | 2 | 1 | 8 |
| Chen et al., 2020 [15] | Cross sectional | 1 | 1 | 1 | 2 | 0 | 1 | 1 | 7 |
| Commodari et al., 2020 [16] | Cross sectional | 1 | 1 | 0 | 2 | 0 | 1 | 1 | 6 |
| Conti et al., 2020 [17] | Longitudinal | 1 | 1 | 0 | 2 | 1 | 2 | 1 | 8 |
| Cusinato et al., 2020 [18] | Cross sectional | 1 | 1 | 0 | 2 | 1 | 2 | 1 | 8 |
| Di Giorgio et al., 2020 [19] | Cross sectional | 1 | 1 | 1 | 2 | 0 | 2 | 1 | 8 |
| Ezpeleta et al., 2020 [20] | Longitudinal | 1 | 1 | 1 | 2 | 1 | 1 | 1 | 8 |
| Francisco et al., 2020 [21] | Cross sectional | 1 | 1 | 0 | 2 | 0 | 1 | 1 | 6 |
| Giannopoulou et al., 2021 [22] | Cross sectional | 1 | 1 | 1 | 2 | 0 | 2 | 1 | 8 |
| Gimenez-Dasi et al., 2020 [23] | Longitudinal | 1 | 1 | 1 | 2 | 1 | 1 | 1 | 8 |
| Graell et al., 2020 [24] | Cross sectional | 0 | 1 | 0 | 2 | 2 | 1 | 1 | 7 |
| Idoiaga et al., 2020a [25] | Cross sectional | 1 | 1 | 0 | 2 | 0 | 1 | 1 | 6 |
| Idoiaga et al., 2020b [26] | Cross sectional | 1 | 1 | 1 | 2 | 0 | 1 | 1 | 7 |
| Kılınçel et al., 2020 [27] | Cross sectional | 0 | 1 | 0 | 2 | 0 | 2 | 1 | 6 |
| Larsen et al., 2020 [28] | Longitudinal | 1 | 1 | 0 | 2 | 0 | 1 | 1 | 6 |
| Lecuelle et al., 2020 [29] | Longitudinal | 1 | 1 | 0 | 2 | 2 | 2 | 1 | 9 |
| Liang et al., 2020 [30] | Cross sectional | 1 | 1 | 0 | 2 | 1 | 1 | 1 | 7 |
| Liebana-Presa et al., 2020 [31] | Cross sectional | 1 | 1 | 0 | 2 | 1 | 2 | 1 | 8 |
| Liu et al., 2020 [32] | Cross sectional | 1 | 1 | 1 | 2 | 1 | 1 | 1 | 8 |
| Magson et al., 2020 [33] | Longitudinal | 1 | 1 | 0 | 2 | 1 | 2 | 1 | 8 |
| Majeed et al., 2020 [34] | Cross sectional | 1 | 1 | 0 | 2 | 0 | 0 | 1 | 5 |
| Mallik et al., 2021 [35] | Cross sectional | 0 | 1 | 0 | 2 | 1 | 1 | 1 | 6 |
| Morgul et al., 2020 [37] | Cross sectional | 0 | 1 | 0 | 2 | 0 | 1 | 1 | 5 |
| Mourouvaye et al., 2020 [38] | Longitudinal | 0 | 1 | 1 | 2 | 0 | 0 | 1 | 5 |
| Nonweilier et al., 2020 [39] | Cross sectional | 1 | 1 | 0 | 2 | 1 | 2 | 1 | 8 |
| Orgilés et al., 2020 [40] | Cross sectional | 1 | 1 | 0 | 2 | 1 | 1 | 1 | 7 |
| Patra et al., 2020 [41] | Cross sectional | 0 | 1 | 0 | 2 | 0 | 1 | 0 | 4 |
| Pisano et al., 2020 [42] | Cross sectional | 1 | 1 | 1 | 2 | 0 | 1 | 0 | 6 |
| Pons et al., 2020 [43] | Cross sectional | 1 | 1 | 1 | 2 | 1 | 1 | 1 | 8 |
| Radwan et al., 2020 [44] | Cross sectional | 1 | 1 | 1 | 2 | 0 | 0 | 1 | 6 |
| Ren et al., 2020 [45] | Cross sectional | 1 | 1 | 1 | 2 | 0 | 1 | 1 | 7 |
| Romero et al., 2020 [46] | Longitudinal | 1 | 1 | 0 | 2 | 0 | 2 | 1 | 7 |
| Sama et al., 2020 [47] | Cross sectional | 1 | 1 | 1 | 2 | 0 | 1 | 0 | 6 |
| Saurabh et al., 2020 [48] | Cross sectional | 0 | 1 | 0 | 2 | 2 | 1 | 1 | 7 |
| Shah et al., 2020a [50] | Cross sectional | 1 | 1 | 1 | 2 | 0 | 1 | 1 | 7 |
| Shah et al., 2020b [49] | Longitudinal | 1 | 0 | 0 | 2 | 0 | 1 | 1 | 5 |
| Smirni et al., 2020 [51] | Cross sectional | 0 | 1 | 0 | 2 | 1 | 2 | 1 | 7 |
| Spinelli et al., 2020 [52] | Cross sectional | 1 | 1 | 0 | 2 | 0 | 2 | 1 | 7 |
| Tang et al., 2020 [53] | Cross sectional | 1 | 1 | 1 | 2 | 0 | 2 | 1 | 8 |
| Troncone et al., 2020 [54] | Cross sectional | 1 | 1 | 0 | 2 | 2 | 2 | 1 | 9 |
| Waite et al., 2020 [55] | Longitudinal | 1 | 1 | 0 | 2 | 0 | 1 | 1 | 6 |
| Wiguna et al., 2020 [56] | Cross sectional | 1 | 1 | 0 | 2 | 0 | 2 | 1 | 7 |
| Xiang et al., 2020 [57] | Longitudinal | 1 | 1 | 1 | 2 | 0 | 2 | 1 | 8 |
| Xie et al., 2020 [58] | Cross sectional | 1 | 1 | 1 | 2 | 0 | 2 | 1 | 8 |
| Yeasmin et al., 2020 [59] | Cross sectional | 0 | 1 | 1 | 2 | 0 | 2 | 1 | 7 |
| Yue et al., 2020 [60] | Cross sectional | 1 | 1 | 0 | 2 | 1 | 1 | 1 | 7 |
| Zhang et al., 2020 [61] | Longitudinal | 1 | 1 | 1 | 2 | 0 | 2 | 1 | 8 |
| Zijlmans et al., 2020 [62] | Cross sectional | 1 | 1 | 0 | 2 | 1 | 1 | 1 | 7 |

**eMethods 1:** Literature search terms

The search terms used were as follows: (“teen*” OR “adolesc*” OR “p?ediatric” OR “child*” OR “young people” OR “student” OR “kid”) AND (“coronavirus” OR “COVID-19” OR “corona*” OR “pandemi*” OR "CoV-19" OR "SARS-CoV-2" OR "2019 nCoV" OR "2019nCoV" OR "2019 novel coronavirus" OR "COVID 19" OR "new coronavirus" OR "novel coronavirus" OR "SARS CoV-2" OR "2019-nCoV") AND (“lockdown” OR “home confinement” OR “quarantin*” OR “self isolation”).

**eMethods 2: Newcastle-Ottawa Scale (NOS) adapted for cross-sectional studies**

The following questions were considered in the Newcastle-Ottawa Scale was adapted for cross-sectional studies (Modesti et al., 2016) [36]

**Selection: (Maximum 5 stars)**

**1) Representativeness of the sample:**

1. Truly representative of the average in the target population. * (all subjects or random sampling)
2. Somewhat representative of the average in the target population. * (nonrandom sampling) c) Selected group of users.
3. No description of the sampling strategy.

**2) Sample size:**

1. Justified and satisfactory. *
2. Not justified.

**3) Non-respondents**:

1. Comparability between respondents and non-respondents characteristics is established, and the response rate is satisfactory. *
2. The response rate is unsatisfactory, or the comparability between respondents and non-respondents is unsatisfactory.
3. No description of the response rate or the characteristics of the responders and the non-responders.

**4) Ascertainment of the exposure (risk factor):**

1. Validated measurement tool. **
2. Non-validated measurement tool, but the tool is available or described.*
3. No description of the measurement tool.

**Comparability: (Maximum 2 stars)**

**1) The subjects in different outcome groups are comparable, based on the study design or analysis. Confounding factors are controlled.**

1. The study controls for the most important factor (select one). *
2. The study control for any additional factor. *

**Outcome: (Maximum 3 stars)**

**1) Assessment of the outcome:**

1. Independent blind assessment. **
2. Record linkage. **
3. Self report. *
4. No description.

**2) Statistical test:**

1. The statistical test used to analyze the data is clearly described and appropriate, and the measurement of the association is presented, including confidence intervals and the probability level (p value). *
2. The statistical test is not appropriate, not described or incomplete.

This scale has been adapted from the Newcastle-Ottawa Quality Assessment Scale for cohort studies to perform a quality assessment of cross-sectional studies for the systematic review, “Are Healthcare Workers’ Intentions to Vaccinate Related to their Knowledge, Beliefs and Attitudes? A Systematic Review”. We have not selected one factor that is the most important for comparability, because the variables are not the same in each study. Thus, the principal factor should be identified for each study. In our scale, we have specifically assigned one star for self-reported outcomes, because our study measures the intention to vaccinate. Two stars are given to the studies that assess the outcome with independent blind observers or with vaccination records, because these methods measure the practice of vaccination, which is the result of true intention.

References:

1. Abawi O, Welling MS, van den Eynde E, van Rossum EFC, Halberstadt J, van den Akker ELT, van der Voorn B (2020) COVID-19 related anxiety in children and adolescents with severe obesity: A mixed-methods study. Clin Obes 10:e12412

2. Abdulah DM, Abdulla BMO, Liamputtong P (2020) Psychological response of children to home confinement during COVID-19: A qualitative arts-based research. Int J Soc Psychiatry:20764020972439

3. Achterberg M, Dobbelaar S, Boer OD, Crone EA (2021) Perceived stress as mediator for longitudinal effects of the COVID-19 lockdown on wellbeing of parents and children. Sci Rep 11:2971

4. Adıbelli D, Sümen A (2020) The effect of the coronavirus (COVID-19) pandemic on health-related quality of life in children. Child Youth Serv Rev 119:105595

5. Alves JM, Yunker AG, DeFendis A, Xiang AH, Page KA (2020) Prenatal exposure to gestational diabetes is associated with anxiety and physical inactivity in children during COVID-19. Clinical Obesity

6. Amorim R, Catarino S, Miragaia P, Ferreras C, Viana V, Guardiano M (2020) The impact of COVID-19 on children with autism spectrum disorder. Rev Neurol 71:285-291

7. Asanov I, Flores F, McKenzie D, Mensmann M, Schulte M (2021) Remote-learning, time-use, and mental health of Ecuadorian high-school students during the COVID-19 quarantine. World development 138:105225

8. Baptista AS, Prado IM, Perazzo MF, Pinho T, Paiva SM, Pordeus IA, Serra-Negra JM (2021) Can children's oral hygiene and sleep routines be compromised during the COVID-19 pandemic? International Journal of Paediatric Dentistry 31:12-19

9. Bentenuto A, Mazzoni N, Giannotti M, Venuti P, de Falco S (2020) Psychological impact of Covid-19 pandemic in Italian families of children with neurodevelopmental disorders. Research in Developmental Disabilities 109:103840-103840

10. Bignardi G, Dalmaijer ES, Anwyl-Irvine AL, Smith TA, Siugzdaite R, Uh S, Astle DE (2020) Longitudinal increases in childhood depression symptoms during the COVID-19 lockdown. Arch Dis Child

11. Cauberghe V, Van Wesenbeeck I, De Jans S, Hudders L, Ponnet K (2020) How Adolescents Use Social Media to Cope with Feelings of Loneliness and Anxiety During COVID-19 Lockdown. Cyberpsychol Behav Soc Netw

12. Çetin FH, Uçar HN, Türkoğlu S, Kahraman EM, Kuz M, Güleç A (2020) Chronotypes and trauma reactions in children with ADHD in home confinement of COVID-19: full mediation effect of sleep problems. Chronobiol Int 37:1214-1222

13. Chen F, Zheng D, Liu J, Gong Y, Guan Z, Lou D (2020) Depression and anxiety among adolescents during COVID-19: A cross-sectional study. Brain Behav Immun 88:36-38

14. Chen IH, Chen CY, Pakpour AH, Griffiths MD, Lin CY (2020) Internet-Related Behaviors and Psychological Distress Among Schoolchildren During COVID-19 School Suspension. J Am Acad Child Adolesc Psychiatry 59:1099-1102.e1091

15. Chen S, Cheng Z, Wu J (2020) Risk factors for adolescents' mental health during the COVID-19 pandemic: a comparison between Wuhan and other urban areas in China. Global Health 16:96

16. Commodari E, La Rosa VL (2020) Adolescents in Quarantine During COVID-19 Pandemic in Italy: Perceived Health Risk, Beliefs, Psychological Experiences and Expectations for the Future. Frontiers in Psychology 11

17. Conti E, Sgandurra G, De Nicola G, Biagioni T, Boldrini S, Bonaventura E, Buchignani B, Della Vecchia S, Falcone F, Fedi C, Gazzillo M, Marinella G, Mazzullo C, Micomonaco J, Pantalone G, Salvati A, Sesso G, Simonelli V, Tolomei G, Troiano I, Cioni G, Masi G, Muratori F, Milone A, Battini R (2020) Behavioural and Emotional Changes during COVID-19 Lockdown in an Italian Paediatric Population with Neurologic and Psychiatric Disorders. Brain Sciences 10

18. Cusinato M, Iannattone S, Spoto A, Poli M, Moretti C, Gatta M, Miscioscia M (2020) Stress, Resilience, and Well-Being in Italian Children and Their Parents during the COVID-19 Pandemic. International journal of environmental research and public health 17

19. Di Giorgio E, Di Riso D, Mioni G, Cellini N (2020) The interplay between mothers' and children behavioral and psychological factors during COVID-19: an Italian study. European child & adolescent psychiatry

20. Ezpeleta L, Navarro JB, de la Osa N, Trepat E, Penelo E (2020) Life Conditions during COVID-19 Lockdown and Mental Health in Spanish Adolescents. Int J Environ Res Public Health 17

21. Francisco R, Pedro M, Delvecchio E, Espada JP, Morales A, Mazzeschi C, Orgilés M (2020) Psychological Symptoms and Behavioral Changes in Children and Adolescents During the Early Phase of COVID-19 Quarantine in Three European Countries. Front Psychiatry 11:570164

22. Giannopoulou I, Efstathiou V, Triantafyllou G, Korkoliakou P, Douzenis A (2021) Adding stress to the stressed: Senior high school students' mental health amidst the COVID-19 nationwide lockdown in Greece. Psychiatry research 295:113560-113560

23. Gimenez-Dasi M, Quintanilla L, Lucas-Molina B, Sarmento-Henrique R (2020) Six Weeks of Confinement: Psychological Effects on a Sample of Children in Early Childhood and Primary Education. Frontiers in Psychology 11

24. Graell M, Morón-Nozaleda MG, Camarneiro R, Villaseñor Á, Yáñez S, Muñoz R, Martínez-Núñez B, Miguélez-Fernández C, Muñoz M, Faya M (2020) Children and adolescents with eating disorders during COVID-19 confinement: Difficulties and future challenges. Eur Eat Disord Rev 28:864-870

25. Idoiaga Mondragon N, Berasategi Sancho N, Dosil Santamaria M, Eiguren Munitis A (2021) Struggling to breathe: a qualitative study of children's wellbeing during lockdown in Spain. Psychol Health 36:179-194

26. Idoiaga N, Berasategi N, Eiguren A, Picaza M (2020) Exploring Children's Social and Emotional Representations of the COVID-19 Pandemic. Front Psychol 11:1952

27. Kılınçel Ş, Kılınçel O, Muratdağı G, Aydın A, Usta MB (2020) Factors affecting the anxiety levels of adolescents in home-quarantine during COVID-19 pandemic in Turkey. Asia Pac Psychiatry:e12406

28. Larsen L, Helland MS, Holt T (2020) The Impact of School Closure and Social Isolation on Children in Vulnerable Families during COVID-19: A Focus on Children’s Reactions.

29. Lecuelle F, Leslie W, Huguelet S, Franco P, Putois B (2020) Did the COVID-19 lockdown really have no impact on young children's sleep? Journal of clinical sleep medicine : JCSM : official publication of the American Academy of Sleep Medicine 16:2121-2121

30. Liang Z, Delvecchio E, Buratta L, Mazzeschi C (2020) "Ripple effect": Psychological responses and coping strategies of Italian children in different COVID-19 severity areas. Revista De Psicologia Clinica Con Ninos Y Adolescentes 7:49-58

31. Liébana-Presa C, Martínez-Fernández MC, Benítez-Andrades JA, Fernández-Martínez E, Marqués-Sánchez P, García-Rodríguez I (2020) Stress, Emotional Intelligence and the Intention to Use Cannabis in Spanish Adolescents: Influence of COVID-19 Confinement. Front Psychol 11:582578

32. Liu Q, Zhou Y, Xie X, Xue Q, Zhu K, Wan Z, Wu H, Zhang J, Song R (2021) The prevalence of behavioral problems among school-aged children in home quarantine during the COVID-19 pandemic in china. Journal of Affective Disorders 279:412-416

33. Magson NR, Freeman JYA, Rapee RM, Richardson CE, Oar EL, Fardouly J (2021) Risk and Protective Factors for Prospective Changes in Adolescent Mental Health during the COVID-19 Pandemic. J Youth Adolesc 50:44-57

34. Majeed S, Ashraf M (2020) Psychological Impacts of Social Distancing During COVID-19 Pandemic in Adolescents of Lahore, Pakistan. Annals of King Edward Medical University Lahore Pakistan 26:165-169

35. Mallik CI, Radwan RB (2021) Impact of lockdown due to COVID-19 pandemic in changes of prevalence of predictive psychiatric disorders among children and adolescents in Bangladesh. Asian J Psychiatr 56:102554

36. Modesti PA, Reboldi G, Cappuccio FP, Agyemang C, Remuzzi G, Rapi S, Perruolo E, Parati G, Settings EWGoCRiLR (2016) Panethnic Differences in Blood Pressure in Europe: A Systematic Review and Meta-Analysis. Plos one 11:e0147601

37. Morgul E, Kallitsoglou A, Essau CA (2020) Psychological effects of the COVID-19 lockdown on children and families in the UK. Revista De Psicologia Clinica Con Ninos Y Adolescentes 7:42-48

38. Mourouvaye M, Bottemanne H, Bonny G, Fourcade L, Angoulvant F, Cohen JF, Ouss L (2020) Association between suicide behaviours in children and adolescents and the COVID-19 lockdown in Paris, France: a retrospective observational study. Archives of disease in childhood

39. Nonweiler J, Rattray F, Baulcomb J, Happe F, Absoud M (2020) Prevalence and Associated Factors of Emotional and Behavioural Difficulties during COVID-19 Pandemic in Children with Neurodevelopmental Disorders. Children-Basel 7

40. Orgilés M, Morales A, Delvecchio E, Mazzeschi C, Espada JP (2020) Immediate Psychological Effects of the COVID-19 Quarantine in Youth From Italy and Spain. Front Psychol 11:579038

41. Patra S, Patro BK, Acharya SP (2020) COVID-19 lockdown and school closure: Boon or bane for child mental health, results of a telephonic parent survey. Asian J Psychiatr 54:102395

42. Pisano L, Galimi D, Cerniglia L (2020) A qualitative report on exploratory data on the possible emotional/behavioral correlates of Covid-19 lockdown in 4-10 years children in Italy.

43. Pons J, Ramis Y, Alcaraz S, Jordana A, Borrueco M, Torregrossa M (2020) Where Did All the Sport Go? Negative Impact of COVID-19 Lockdown on Life-Spheres and Mental Health of Spanish Young Athletes. Frontiers in Psychology 11

44. Radwan E, Radwan A, Radwan W (2020) The role of social media in spreading panic among primary and secondary school students during the COVID-19 pandemic: An online questionnaire study from the Gaza Strip, Palestine. Heliyon 6:e05807-e05807

45. Ren H, He X, Bian X, Shang X, Liu J (2021) The Protective Roles of Exercise and Maintenance of Daily Living Routines for Chinese Adolescents During the COVID-19 Quarantine Period. J Adolesc Health 68:35-42

46. Romero E, López-Romero L, Domínguez-Álvarez B, Villar P, Gómez-Fraguela JA (2020) Testing the Effects of COVID-19 Confinement in Spanish Children: The Role of Parents' Distress, Emotional Problems and Specific Parenting. Int J Environ Res Public Health 17

47. Sama BK, Kaur P, Thind PS, Verma MK, Kaur M, Singh DD (2021) Implications of COVID-19-induced nationwide lockdown on children's behaviour in Punjab, India. Child Care Health Dev 47:128-135

48. Saurabh K, Ranjan S (2020) Compliance and Psychological Impact of Quarantine in Children and Adolescents due to Covid-19 Pandemic. Indian J Pediatr 87:532-536

49. Shah R, Raju VV, Sharma A, Grover S (2020) Impact of COVID-19 and Lockdown on Children with ADHD and Their Families-An Online Survey and a Continuity Care Model. Journal of Neurosciences in Rural Practice

50. Shah S, Kaul A, Shah R, Maddipoti S (2020) Impact of Coronavirus Disease 2019 Pandemic and Lockdown on Mental Health Symptoms in Children. Indian pediatrics

51. Smirni P, Lavanco G, Smirni D (2020) Anxiety in Older Adolescents at the Time of COVID-19. J Clin Med 9

52. Spinelli M, Lionetti F, Pastore M, Fasolo M (2020) Parents' Stress and Children's Psychological Problems in Families Facing the COVID-19 Outbreak in Italy. Frontiers in Psychology 11

53. Tang S, Xiang M, Cheung T, Xiang YT (2021) Mental health and its correlates among children and adolescents during COVID-19 school closure: The importance of parent-child discussion. J Affect Disord 279:353-360

54. Troncone A, Chianese A, Zanfardino A, Cascella C, Piscopo A, Borriello A, Rollato S, Casaburo F, Testa V, Iafusco D (2020) Disordered eating behaviors in youths with type 1 diabetes during COVID-19 lockdown: an exploratory study. Journal of Eating Disorders 8

55. Waite P, Pearcey S, Shum A, Raw J, Patalay P, Creswell C (2020) How did the mental health of children and adolescents change during early lockdown during the COVID-19 pandemic in the UK?

56. Wiguna T, Anindyajati G, Kaligis F, Ismail RI, Minayati K, Hanafi E, Murtani BJ, Wigantara NA, Putra AA, Pradana K (2020) Brief Research Report on Adolescent Mental Well-Being and School Closures During the COVID-19 Pandemic in Indonesia. Front Psychiatry 11

57. Xiang M, Yamamoto S, Mizoue T (2020) Depressive symptoms in students during school closure due toCOVID-19 inShanghai. Psychiatry and Clinical Neurosciences 74:664-666

58. Xie X, Xue Q, Zhou Y, Zhu K, Liu Q, Zhang J, Song R (2020) Mental Health Status Among Children in Home Confinement During the Coronavirus Disease 2019 Outbreak in Hubei Province, China. JAMA Pediatr 174:898-900

59. Yeasmin S, Banik R, Hossain S, Hossain MN, Mahumud R, Salma N, Hossain MM (2020) Impact of COVID-19 pandemic on the mental health of children in Bangladesh: A cross-sectional study. Child Youth Serv Rev 117:105277

60. Yue J, Zang X, Le Y, An Y (2020) Anxiety, depression and PTSD among children and their parent during 2019 novel coronavirus disease (COVID-19) outbreak in China. Curr Psychol:1-8

61. Zhang L, Zhang D, Fang J, Wan Y, Tao F, Sun Y (2020) Assessment of Mental Health of Chinese Primary School Students Before and After School Closing and Opening During the COVID-19 Pandemic. Jama Network Open 3

62. Zijlmans J, Teela L, van Ewijk H, Klip H, van der Mheen M, Ruisch H, Luijten M, van Muilekom M, Oostrom K, Buitelaar J (2020) Mental and social health of children and adolescents with pre-existing mental or somatic problems during the COVID-19 pandemic lockdown. MedRxiv
